# Antibody–Drug Conjugates in Hematological Malignancies: Current Landscape and Future Perspectives

**DOI:** 10.3390/ijms27021025

**Published:** 2026-01-20

**Authors:** Maria Chiara Montalbano, Matilde Micillo, Silvia Deaglio, Tiziana Vaisitti

**Affiliations:** Functional Genomics Unit, Department of Medical Sciences, University of Turin, 10126 Turin, Italy; mariachiara.montalbano@unito.it (M.C.M.); matilde.micillo@unito.it (M.M.); tiziana.vaisitti@unito.it (T.V.)

**Keywords:** antibody–drug conjugates, payload, linker, hematological malignancies, leukemia, lymphoma

## Abstract

The therapeutic landscape for hematological malignancies has been fundamentally revolutionized over the last decade by the introduction of targeted antibodies. Notably, antibody–drug conjugates (ADCs) have emerged as a critical breakthrough, significantly improving the efficacy of immune-based treatment. ADCs function as highly sophisticated delivery systems: a selective monoclonal antibody recognizes a specific cell-surface target, guiding a potent toxic payload, attached via a chemical linker, directly into the cancer cell upon internalization. Intensive research has been dedicated to optimizing these components—improving antibody selectivity, enhancing linker stability, and utilizing highly effective payloads—which has resulted in a plethora of compounds that have reached patients’ bedsides and improved the clinical course of different tumors. This review provides a crucial overview of the current landscape of approved ADCs for hematological malignancies. It critically discusses their existing limitations and details the essential structural and chemical improvements that have yielded more potent and selective next-generation tools, finally presenting future strategies to generate highly effective “bullets” capable of decisively improving long-term disease prognosis.

## 1. Introduction

The enhanced knowledge of the molecular mechanisms and genetic features of hematological disorders has increasingly enabled the integration of targeted therapies into treatment strategies, reshaping the therapeutic scenario by complementing, or even replacing, conventional chemotherapy. Indeed, systemic chemotherapy, which has largely represented the standard of care during the last century, is associated with off-target toxicities, often limiting the intensity of treatment regimens and restricting the eligible patient population. Therefore, targeted therapies are significantly modifying the prognosis of patients and substantially increasing treatment-eligible cohorts.

In this evolving scenario, monoclonal antibodies (mAbs), comprising both non-modified (naked) and cytotoxic-conjugated structures, play a central role in modern oncology and immunology. Although the technology for antibody–drug conjugates (ADCs) has existed for over four decades, it is only in recent years that they have rapidly and exponentially expanded in large-scale manufacturing and clinical approvals. To date, more than 300 ADCs have been developed, of which 16 were approved globally by 2024 for clinical use in both solid and hematological tumors. In addition, over two hundred compounds are currently under evaluation in clinical trials worldwide [1,2].

This review aims to provide a snapshot of current clinical applications of ADCs in the field of hematological malignancies, outlining their key features and discussing their potential and limitations, with a focus on future improvements. Furthermore, it will explore new frontiers in the development of promising next-generation ADCs to be used for targeted therapies in hemato-oncology.

## 2. ADC Structure and Mechanism of Action

**Figure 1:**
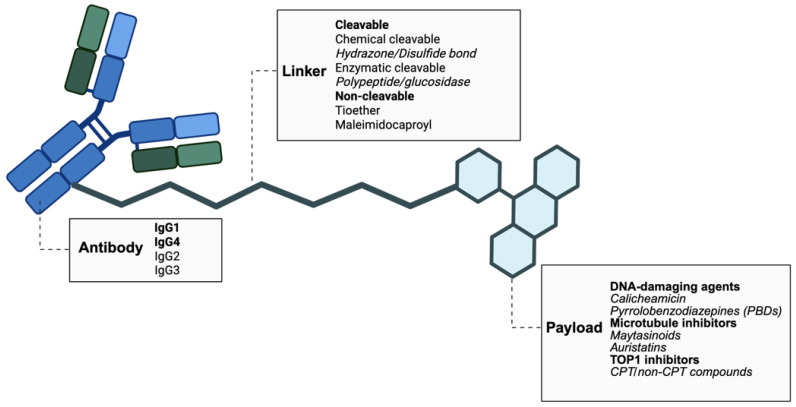
Scheme of antibody–drug conjugate structure. Antibody–drug conjugates (ADCs) are made of an antibody, mainly belonging to the IgG1 and IgG4 class of antibody, conjugated to a payload via either a cleavable or a non-cleavable linker. The main payloads used are listed. This image was created in Biorender by Maria Chiara Montalbano (2025; https://BioRender.com; accessed on 1 December 2025; University Department license).

ADCs are composed of a mAb conjugated to a cytotoxic agent through a chemical linker (Figure 1). They aim to selectively deliver their payload to tumor cells while sparing healthy tissues.

**Figure 2:**
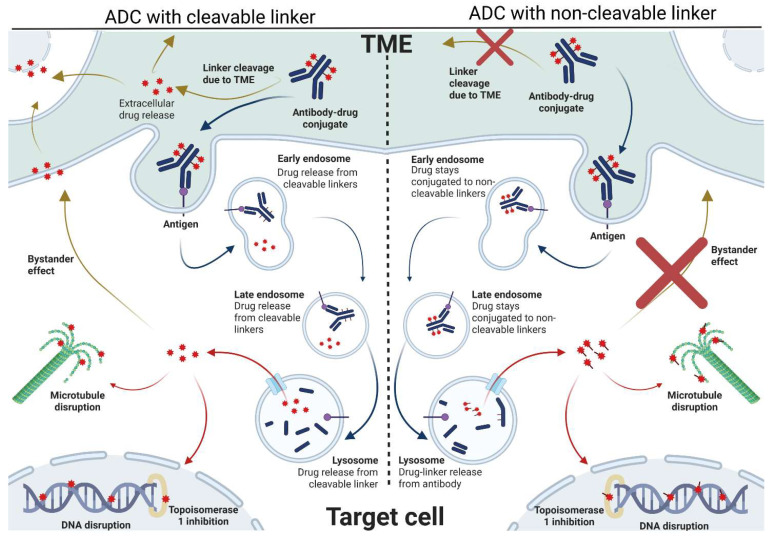
Mechanisms of action of an antibody–drug conjugate. The antibody recognizes a specific antigen/epitope on the target cell surface and, once bound, the complex is internalized with subsequent fusion with lysosomes. The payload is then released within the intracellular space where it is free to act on its specific molecular target, finally inducing cell apoptosis. Depending on the nature of the linker, the payload can either be released outside the target cell because of tumor microenvironment (TME) conditions or as a consequence of tumor cell death, with subsequent activation of the bystander effect (cleavable linker, left panel). In the case of non-cleavable linker, there is no TME-mediated ADC processing and the payload can only be released in the intracellular space, after lysosome processing. The bystander effect is prevented because of the linker chemistry (non-cleavable, right panel). This image was created in Biorender by Matilde Micillo (2026; https://BioRender.com; accessed on 6 January 2026; University Department license).

The ADC mechanism of action follows three main steps: (i) binding of the mAb to the target antigen, preferably a tumor-associated antigen (TAA) expressed on the tumor cell surface; (ii) internalization; and (iii) lysosomal degradation, with consequent intracellular release of the payload, which, in turn, can exert its cytotoxic action (Figure 2).

Besides being merely a carrier, the antibody unit may directly and indirectly interact with the immune system through immunogenic cell death, crystallizable fragment (Fc)-effector functions, Fc-mediated payload uptake, and bystander payload uptake that either kills or stimulates immune cells (Figure 2) [3]. Each component critically shapes the ADC pharmacokinetic (PK) and pharmacodynamic (PD) profile. Over the years, their progressive refinement has driven the evolution of increasingly advanced ADC generations.

Humanized or fully human antibodies represent the predominant backbone of currently available compounds. Among mAbs, the IgG1 subtype is by far the most widely used in ADC therapeutic applications due to its low immunogenicity and favorable PK properties, followed by IgG4 (Figure 1). Their Fc plays an active role enhancing therapeutic efficacy through interaction with Fc receptors (FcγRs) expressed on the immune cell surface, mediating additional immune-mediated cytotoxic effects, mainly antibody-dependent cellular cytotoxicity (ADCC), antibody-dependent cellular phagocytosis (ADCP), and complement-dependent cytotoxicity (CDC) [2,4]. Improved antibody stability is warranted by neonatal Fc receptor (FcRn) binding, preventing antibody degradation and ensuring its persistence at the target site, extending its half-life [2].

Antibody-ideal targets should be, if not exclusively, a molecule expressed more on tumor cells compared to normal tissues to minimize undesired toxicity on the healthy counterpart. Moderate binding affinity is often preferred, ensuring robust interaction with highly expressing tumor cells, avoiding binding to normal tissues with low antigen density [5].

The linker ensures conjugation of the payload to the antibody, yet its role extends well beyond simple chemical attachment, influencing drug efficacy and safety. Linker stability and cleavability are key determinants of the payload release mechanism. Linkers are designed to be either cleavable or non-cleavable. The former enable payload release either following endosomal or lysosomal processing or in response to enzymatic or chemical reactions, such as protease activity, acidic pH, or redox conditions, occurring within the tumor microenvironment (TME) [6]. Consequently, cleavable linkers can mediate a “bystander effect” through extracellular payload diffusion, with dual implications: while it may increase off-target toxicity, it also enhances antitumor efficacy by eliminating tumor-surrounding cells characterized by a low or heterogeneous antigen expression [5,7]. In contrast, non-cleavable linkers require complete lysosomal degradation of the antibody to release the cytotoxic payload in the intracellular space, thereby minimizing extracellular payload dissemination and reducing bystander-associated toxicity [8] (Figure 2).

Linker stability remains a central challenge in ADC development. To date, no approved ADC features a completely stable linker, with most of them undergoing substantial deconjugation due to TME conditions or antibody–linker dissociation driven by the conjugation chemistry [1,6]. The consequence is that only ~50% of the drug is linked to the antibody structure in the circulation one week after administration [1]. Among the currently approved ADCs for hematological malignancies (Table 1), different linker chemistries have been exploited. Brentuximab vedotin and polatuzumab vedotin (Pola-V) are made with a valine–citrulline linker, while the valine–alanine linker is used for loncastuximab tesirine, all protease-cleavable linkers sensitive to lysosomal enzymes [9,10,11]. Alternatively, acid-cleavable linkers, such as hydrazones, which remain relatively stable at physiological pH but undergo hydrolysis in acidic compartments, have been employed in gemtuzumab ozogamicin (GO) and inotuzumab ozogamicin (InO). Of note, acid-sensitive linkers have been associated with a higher risk of premature payload release in the circulation [12,13,14]. Lastly, belantamab mafodotin is made with a non-cleavable linker, maleimidocaproyl, that can only be processed within the lysosomal compartment, allowing for payload release [15].

A wide range of cytotoxic payloads have been investigated over the course of ADC development. Early-generation ADCs employed conventional chemotherapeutic agents such as taxanes, methotrexate, and anthracyclines, yet their efficacy was poor due to inefficient delivery to tumor cells [7]. This experience led to the concept that the payload needs to exhibit strong cytotoxic activity at very low concentrations, ideally achieving 50% inhibition (IC_50_) in the low or even sub-nanomolar range. Indeed, modern ADCs incorporate significantly more potent payloads than traditional systemic chemotherapies, limiting their use as standalone therapeutic agents. Currently, among ADCs approved for clinical use, five classes of payloads are employed, showing different mechanisms of actions: DNA damaging agents, microtubules inhibitors and, more recently, topoisomerase 1 (TOP1) inhibitors (Figure 1) [1].

DNA-damaging agents, such as calicheamicin and pyrrolobenzodiazepines (PBDs), induce cell death by binding to the minor groove of double-strand DNA, causing its disruption. Calicheamicin was the first payload conjugated to a mAb that received approval from the Food and Drug Administration (FDA), GO, for the treatment of acute myeloid leukemia (AML; Table 1) [34]. PBDs show enhanced cytotoxic efficacy, covalently binding to the DNA strands via interstrand DNA cross-linking, without inducing significant structural distortions, with reduced recognition by the cellular DNA repair machinery [2]. However, their systemic toxicity initially limited their application, later restricting their use mainly to targeted therapeutic strategies in the setting of hematological malignancies. Loncastuximab tesirine represents the only approved member of this family of payloads, employed in the treatment of relapsed or refractory (R/R) diffuse large B-cell lymphoma (DLBCL; Table 1) [35].

Microtubule inhibitors, including auristatins and maytasinoids, act by inhibiting microtubule polymerization. Auristatins are a class of synthetic compounds whose use as payloads in ADC synthesis found application in both solid tumors and hematological malignancies. Monomethyl auristatin E (MMAE) and monomethyl auristatin F (MMAF) are the most exploited members of this family. MMAE is incorporated in BV and Pola-V, two well-known FDA-approved ADC drugs (Table 1). DM1 and DM4 represent the most widely used maytasinoid compounds. Trastuzumab emtansine has been the first ADC approved for the treatment of solid tumors, in particular HER2-positive breast cancer, incorporating DM1 as cytotoxic payload [1,7].

Lastly, TOP1 inhibitors are the most recently approved class of ADC payloads. Their distinguishing feature lies in their intermediate cytotoxicity compared to the other compounds discussed above. The potential development of this class of payload has been allowed by recent structural modifications that have led to the design of highly loaded ADCs, with a greater number of payload molecules per antibody, and a consequent increased drug-to-antibody ratio (DAR) [7]. Topoisomerases are nuclear enzymes involved in DNA repair. Topoisomerase 1 cleaves single-stranded DNA, while isoform 2 targets double-stranded DNA. The inhibition of these enzymes prevents their interaction with DNA, leading to the accumulation of unrepaired genetic damage and subsequent induction of apoptosis. TOP1 inhibitors are mainly classified into two groups: camptothecin (CPT) derivatives and non-CPT compounds. To date, two CPT-based derivatives have been conjugated to mAbs and approved for clinical use: deruxtecan (DXd) and SN-38. Two other notable compounds belonging to this class of payloads are exatecan, characterized by greater hydrophilicity, and irinotecan, a prodrug version of the SN38, exhibiting lower hydrophobicity and reduced toxicity. Non-CPT derivatives are currently undergoing early-phase evaluation as small molecules: compared to CPT compounds, they exhibit greater stability and activity, along with a higher cytotoxicity profile [7].

## 3. ADCs in the Landscape of Hematological Malignancies: Overview of the Approved Compounds

Hematological malignancies represent the first therapeutic area where ADCs were applied, marked by the approval of GO in 2000 for the treatment of AML. Since then, six compounds have been approved by the FDA and the European Medicine Agency (EMA) (Table 1).

This section of the review is organized to present the approved ADCs in the hematological field, their clinical applications, and the results obtained from clinical trials.

### 3.1. Gemtuzumab Ozogamicin

Gemtuzumab ozogamicin (GO, Mylotarg^®^) is made of an anti-CD33 humanized IgG4k antibody covalently bound to the cytotoxic payload N-acetyl-gamma-calicheamicin. CD33 is a transmembrane glycoprotein expressed on myeloid cell lineages, with a higher expression characterizing AML blasts.

GO received accelerated FDA approval in 2000 based on three trials demonstrating efficacy of GO used as single agent in patients with R/R AML [12,34]. However, the randomized phase 3 SWOG S0106 and AML-17 trials, which evaluated the addition of GO to standard chemotherapy in two different aged cohorts of patients with newly diagnosed AML, failed to demonstrate clinical benefit compared to the control arms. Both studies also reported increased induction-related mortality, leading to the withdrawal of GO from the market a decade later [36,37]. Subsequent evidence emerged from two large randomized controlled trials (RCTs), AML15 and AML16, which employed a reduced and fractionated dose of GO corresponding to the approved 3 mg/m^2^ dose. These studies demonstrated improved overall survival (OS) in patients with core-binding factor (CBF) AML (AML15) and improved OS with reduced 3-year cumulative incidence of relapse (AML16) [38,39]. The addition of GO to chemotherapy was also evaluated by the MD Anderson Cancer Center (MDACC) group that analyzed outcomes in patients treated with the FLAG (fludarabine, cytarabine, G-CSF) regimen combined with GO. Favorable results were observed in patients with CBF AML, with improvements in both OS and 3-year relapse-free survival (RFS) [40]. Further support came from the pivotal French phase 3 RCT ALFA-0701 (MF-3), in which GO was administered with the currently approved schedule (days 1, 4, and 7 during induction, and day 1 of each consolidation cycle). This trial showed significant improvement in event-free survival (EFS) and OS at 2 years. Consistent with AML15 and MDACC findings, the survival benefit of GO was observed primarily in patients with favorable and intermediate cytogenetic risk profiles [41]. Finally, in a large meta-analysis of a cohort of more than 3000 patients from five RCTs, GO was shown to significantly improve 5-year OS and reduce the risk of relapse. The absolute survival benefit was registered especially in patients with favorable cytogenetic characteristics [42]. Collectively, these data led to the reapproval of GO by the FDA in 2017, followed by EMA in the subsequent year [43,44,45].

Considering the evidence discussed above, the European LeukemiaNet (ELN) guidelines recommend the use of GO for the treatment of CD33^+^ AML with favorable cytogenetic risk [46].

### 3.2. Brentuximab Vedotin

Brentuximab vedotin (BV, Adcetris^®^) consists of a humanized anti-CD30 IgG1 antibody conjugated to the cytotoxic payload MMAE via a highly stable cleavable linker. CD30 is a transmembrane glycoprotein belonging to the tumor necrosis factor (TNF) receptor family. Its downstream signaling cascade promotes cellular proliferation and inhibits apoptosis through the NF-κB pathway [47,48]. CD30 expression is highest on Reed–Sternberg cells and in systemic anaplastic large-cell lymphoma (sALCL), whereas it is lower and more variable in other lymphoma subtypes, including peripheral and cutaneous T-cell lymphomas (PTCL and CTCL). In normal tissues, CD30 expression is limited, except for activated lymphocytes [47,49].

In the last decade, BV has significantly influenced the treatment paradigm of classical Hodgkin lymphoma (cHL). In 2018, based on promising results from preliminary phase 1/2 studies, the phase 3 ECHELON-1 trial was conducted to compare BV combined with doxorubicin, vinblastine, and dacarbazine (BV-AVD) to the standard ABVD (doxorubicin, vinblastine, bleomycin, dacarbazine) regimen in treatment-naïve (TN) patients with stage III/IV cHL. The experimental arm showed significant superior modified progression-free survival (mPFS) (82.1% vs. 77.2%), resulting in regulatory approval [50]. A significant improvement in OS and PFS with BV-AVD compared to ABVD has recently been reported in the ECHELON-1 trial median follow-up of approximately six years, which led to a shift in clinical practice largely replacing ABVD in this scenario [50]. In the first line setting, the European Commission recently approved the BrECADD regimen (BV combined with reduced doses of etoposide, cyclophosphamide, and increased doses of doxorubicin, dacarbazine, and dexamethasone) for adult patients with stage IIb with risk factors/III or IV cHL, based on the results of the HD21 trial. The study demonstrated both superior PFS and the improved tolerability of BrECADD compared to escalated BEACOPP (bleomycin, etoposide, adriamycin, cyclophosphamide, vincristine, procarbazine, prednisone) in the context of intensified systemic chemotherapy [51]. The use of BV has also recently been extended to both pediatric and elderly patients. In 2022, the FDA approved BV in combination with the AVEPC regimen (doxorubicin, vincristine, etoposide, prednisone, and cyclophosphamide) for patients older than 2 years with high-risk cHL. This approval was based on the results of the AHOD1331 study, which demonstrated the superior efficacy of adding BV in terms of EFS without significant differences in toxicity [52]. Furthermore, BV was also administered sequentially before and after AVD in untreated patients with cHL older than 60 years, showing significant efficacy and tolerability even in this patient population [53].

In the R/R setting, BV found its first indication and approval. Phase 2 trials conducted in R/R cHL after autologous stem cell transplantation (ASCT) and in R/R sALCL showing high response rates led to accelerated FDA approval in 2011, followed shortly thereafter by EMA [19,54,55,56]. In 2015, the indication was converted to full approval for use as post-ASCT maintenance therapy in patients with cHL at high risk of relapse, based on the results of the phase 3 AETHERA trial, in which a benefit in terms of PFS in patients treated with BV compared to placebo was demonstrated across all risk groups [57].

In addition to its role in the treatment of cHL, BV is also currently approved for the treatment of sALCL and R/R CD30^+^ CTCL. The ECHELON-2 trial, which enrolled newly diagnosed CD30^+^ PTCL patients—mainly sALCL, randomized to receive BV plus CHP (cyclophosphamide, doxorubicin, and prednisone) or CHOP (cyclophosphamide, doxorubicin, vincristine and prednisone)—demonstrated significantly improved PFS and OS with the BV-CHP regimen [58,59]. Current guidelines recommend BV in combination with CHP for TN adults with sALC and its use for R/R cases. BV is also approved for CD30^+^ CTCL patients who have received at least one prior systemic therapy, based on results from the phase 3 ALCANZA study. In this trial, patients with MF or primary cutaneous CD30^+^ lymphoma were randomized to BV or physician’s choice. The BV arm showed significant superiority in terms of objective responses compared to the control arm [60].

Finally, considering the variable expression of CD30^+^ in lymphoproliferative disorders, the use of BV has recently been extended to other malignancies, mainly LBCL. In this setting, the combination of BV with chemoimmunotherapy (CIT) has demonstrated significant efficacy, leading to the recent FDA approval of BV in combination with lenalidomide and rituximab for R/R patients ineligible for ASCT or CAR-T therapy, based on the results of the ECHELON-3 trial [61].

### 3.3. Inotuzumab Ozogamicin

Inotuzumab (InO, Besponsa^®^) is an anti-CD22 directed humanized IgG4 antibody conjugated to N-acetyl-gamma-calicheamicin through a cleavable linker [62]. CD22 is a transmembrane receptor expressed on the B-cell surface and by most B-cell malignancies, playing an inhibitory role in B-cell receptor (BCR) signaling [63].

Early phase 2 trials of InO in B-cell non-Hodgkin lymphomas (NHLs) yielded encouraging results, even though phase 3 studies failed to confirm the data, leading to discontinuation of its development in this disease setting [62,64,65,66].

Based on preclinical evidence indicating the higher sensitivity of acute lymphoblastic leukemia (ALL) cells to calicheamicin compared with NHL, a phase 2 trial was subsequently conducted in patients with R/R ALL. The study enrolled 90 patients, obtaining a 58% complete remission (CR) rate and demonstrating a fractionated dosing schedule efficacy and reduced toxicity compared with the single-dose regimen [67,68].

InO was further evaluated in a multicenter phase 1/2 trial, which established 1.8 mg/m^2^ as the recommended dose, administered fractionally over a 28-day cycle. Among 72 patients with R/R B-cell ALL, the CR/CRi (CR with incomplete marrow recovery) rate was 68%, with 84% of responders achieving measurable residual disease (MRD) negativity [23]. Based on these findings, the pivotal phase 3 INO-VATE trial compared InO to standard chemotherapy in the salvage setting [69]. InO significantly improved CR/CRi rates (81% vs. 29%), MRD negativity (78% vs. 28%) and PFS (5.0 vs. 1.8 months). At 3 years, OS remained higher with InO (20% vs. 7%), and a greater proportion of patients proceeded to hematopoietic stem cell transplantation (HSCT; 48% vs. 22%) [70,71]. These data led to the regulatory approval of InO from FDA ad EMA for adults with R/R B-cell ALL [72]. InO is also now increasingly used to bridge patients to HSCT and to eliminate MRD [63].

Following the positive outcomes of InO as a salvage monotherapy, clinical studies evaluated its combination with low-intensity chemotherapy and targeted agents in both R/R ALL and frontline setting. These studies focused on both elderly patients, whose treatment remains challenging, and younger cohorts.

The integration of InO into the mini-hyper-CVD regimen (cyclophosphamide, vincristine, dexamethasone plus methotrexate and cytarabine) in patients with R/R ALL led to global responses reaching 78% in the treated population and an overall MRD negativity rate of 82% among responders. Notably, 44% of patients proceeded to HSCT, supporting the potential of this combination. The addition of the bispecific antibody blinatumomab in subsequent studies demonstrated improved results, even in the first-line setting of an elderly patient subgroup [70,71,72,73,74]. A clinical trial is currently ongoing to compare this regimen with the standard adjusted-dose hyper-CVAD in patients over 66 years of age with newly diagnosed B-ALL [72]. A 3-year OS rate of 88% was also achieved from MDACC, which assessed the combination of InO and blinatumomab with the hyper-CVAD regimen alternating with high-dose methotrexate and cytarabine in patients younger than 60 years [62,72]. The INITIAL-1 and EWALL-InO trials tested InO combinations with dexamethasone or chemotherapy, respectively, as induction therapy in newly diagnosed ALL patients aged 55 years or older, reporting high response rates. Finally, the Alliance A041703 trial was the first to investigate a completely chemotherapy-free frontline approach in elderly patients with ALL, combining InO with blinatumomab [62,72,75,76].

InO has also demonstrated promising activity in R/R Philadelphia chromosome-positive (Ph^+^) B-ALL and Ph-like ALL. Different trials evaluating its efficacy in subgroup analyses both as a single agent or in combination with tyrosine kinase inhibitors (TKIs) are ongoing, showing preliminary but encouraging response rates [62,77,78]. Currently, the use of InO in adult patients with Ph^+^ ALL is restricted to those who have failed prior TKI therapy [79].

### 3.4. Polatuzumab Vedotin

Polatuzumab vedotin (Pola-V, Polivy^®^) is composed of a humanized IgG1 antibody targeting CD79b, covalently linked to MMAE via a cleavable linker [27]. CD79b is a signaling component of the BCR, ubiquitously expressed on the surface of B cells and mature B-cell lymphoproliferative malignancies [10,80].

First trials of Pola-V in R/R NHL patients, alone or in combination with the anti-CD20 mAb rituximab, showed a modest overall response rate (ORR) and insufficient CR rates, prompting its combination with CIT [28,80,81]. In transplant-ineligible R/R DLBCL patients, a phase 1/2 trial comparing Pola-V combined with bendamustine and rituximab (Pola-BR) versus bendamustine and rituximab (BR) alone demonstrated significantly higher CR rates (40% vs. 17.5%), mPFS (9.5 vs. 3.7 months), and OS (12.4 vs. 4.7 months) [80]. The clinical benefit of the Pola-BR regimen was shown to be independent of the lymphoma cell of origin and its molecular profile [80,82]. The subsequent extension study confirmed efficacy, reporting 41.5% ORR (mainly CR) and a favorable toxicity profile [83]. Based on these results, in 2019, the FDA granted accelerated approval for Pola-BR in R/R DLBCL after at least two prior lines of therapy. The following year, the EMA granted conditional marketing authorization for Pola-BR in patients with transplant-ineligible R/R DLBCL [83]. More recently, Pola-V has been incorporated into the first-line treatment of DLBCL. Its combination with rituximab, cyclophosphamide, doxorubicin, and prednisone in the Pola-R-CHP scheme demonstrated high ORR (89%) and CR (77%) in phase 1b/2 studies, paving the way for the phase 3 POLARIX trial [29]. The comparison with the standard R-CHOP regimen showed a reduced risk of disease progression, relapse, or death (stratified hazard ratio [HR] 0.73) and improved 2-year PFS (76.7% vs. 70.2%) in untreated intermediate- or high-risk DLBCL patients in the experimental arm [84]. The toxicity profile of the two regimens was comparable; the known adverse effects of MMAE, namely, cytopenia and peripheral neuropathy, represented the most concerning issues in patients treated with Pola-V [84]. Based on these data, the Pola-R-CHP regimen obtained full approval for previously untreated adult DLBCL patients. The increasing use of targeted combination therapies prompted the evaluation of Pola-V in combination with bispecific antibodies (bsAbs), demonstrating promising response rates and an acceptable safety profile. Notably, recent promising data have been published about the combination of Pola-V with mosunetuzumab, a CD20/CD3 T-cell-engaging bispecific antibody, in patients with R/R DLBCL ineligible for transplantation in a phase 1b/2 trial [85].

### 3.5. Loncastuximab Tesirine

Loncastuximab tesirine (Zynlonta^®^) consists of a humanized anti-CD19 IgG1 antibody conjugated to the alkylating agent PBD dimer. The high potency of PBD compounds led to the development of ADCs with lower DARs, with loncastuximab tesirine being the only approved compound characterized by a DAR of 2 [7,35,86]. Furthermore, owing to its high stable linker chemistry, loncastuximab tesirine represents the only approved ADC retaining approximately 80% of its conjugated form at the same time point [1].

CD19 is a well-established B-cell-specific marker, belonging to the Ig superfamily and playing a crucial role in the BCR-mediated regulation of intracellular signaling. It is widely expressed on B lymphocytes, from the pre-B stage through to late stages of differentiation [87].

Loncastuximab tesirine clinical activity in R/R DLBCL emerged from the phase 1/2 LOTIS-1/2 trials [30]. In the phase 2 LOTIS-2 trial, loncastuximab tesirine achieved an ORR of 48.3% in heavily pretreated patients. The median duration of response (DOR) was 13.4 months, and durable CRs were observed, with over 30% of patients remaining event-free for ≥1 year. Loncastuximab tesirine was generally well tolerated, with most treatment-emergent adverse events (TEAEs) being manageable and of mild to moderate severity [31,32,88]. Analyses of the LOTIS-2 trial also indicated that both younger and older patients (>70 years) showed comparable outcomes across most assessed parameters [89].

These findings led to the regulatory approval from FDA and EMA of loncastuximab tesirine as a single agent for R/R DLBCL and high-grade B-cell lymphoma (HGBL) after two or more lines of systemic therapy, representing the first ADC with a PBD payload approved for this indication. Ongoing trials are evaluating loncastuximab tesirine in combination with rituximab in R/R DLBCL compared to the standard regimen rituximab, gemcitabine, and oxaliplatin (R-GemOx) and in association with mosunetuzumab in the LOTIS-5 and LOTIS-7 trials [86]. As confirmed by a recent retrospective analysis, loncastuximab tesirine monotherapy currently represents a potential therapeutic option in heavily pretreated and refractory DLBCL patients; however, further investigation will provide greater evidence of its role in the R/R or frontline setting and its potential role in different B-cell histologies [90,91].

### 3.6. Belantamab Mafodotin

Belantamab mafodotin (Blenrep^®^) is made of a humanized, afucosylated anti-BCMA IgG1 antibody conjugated to MMAF. BCMA (also known as CD269), a TNF receptor superfamily member essential for plasma cell survival, has emerged as a critical target in multiple myeloma (MM). The overexpression and activation of BCMA have been associated with MM progression, making it an attractive therapeutic target [92]. However, the clinical development of belantamab mafodotin has been complex, involving multiple phases of approval and subsequent withdrawal [93].

Belantamab mafodotin was initially approved based on the phase 1 and 2 DREAMM-1 and 2 trials, showing 32% ORR in triple-class refractory MM [94,95]. However, the subsequent phase 3 DREAMM-3 trial, comparing belantamab mafodotin monotherapy with the combination of pomalidomide and dexamethasone in patients with R/R multiple myeloma (RRMM) after ≥2 prior lines of therapy, failed to show PFS superiority, leading to its withdrawal from both the US and European markets between 2023 and 2024 [96]. Nevertheless, the therapeutic potential of belantamab mafodotin has continued to be investigated, particularly in combination with immunomodulatory agents (IMIDs) and proteasome inhibitors (PIs). Preclinical studies have demonstrated both synergistic and additive effects, thereby supporting the rationale for its use in combination therapies [93]. In particular, the combination studies DREAMM-7 and DREAMM-8 directly compared belantamab mafodotin plus bortezomib and dexamethasone (BVd) versus daratumumab, bortezomib, and dexamethasone (DREAMM-7), and belantamab mafodotin plus pomalidomide and dexamethasone (BPd) versus pomalidomide, bortezomib, and dexamethasone (DREAMM-8) in patients with RRMM. Both studies extended their follow-up periods to allow for PFS analyses [97,98]. The 3-year analysis from the DREAMM-7 trial demonstrated an OS benefit in patients treated with the BVd triplet. Similarly, the interim analysis from the DREAMM-8 trial presented at EHA 2025 showed improved PFS and a trend toward better OS in patients receiving the experimental regimen, laying the groundwork for the data that supported the recent approval of the drug [98].

Currently, belantamab mafodotin is approved in Europe for the treatment of RRMM in combination with bortezomib and dexamethasone (BVd regimen), as well as in combination with pomalidomide and dexamethasone (BPd regimen) in patients who have previously received a lenalidomide-based line of therapy. In the US market, its license was revoked by the FDA in 2023 and has not yet been reinstated.

## 4. What We Have Learned from the Clinical Trials

The clinical development of ADCs in hematologic malignancies has followed a “learning curve”, ultimately revealing that the efficacy of these agents is not solely determined by target expression or payload cytotoxicity. Indeed, the final clinical efficacy of an ADC is dictated by the complex interplay among drug features (antigen, linker, and payload), patient-related characteristics, dosing strategies, and combination with other therapeutic agents. To this end, large clinical trials are essential to (i) define optimal dosing and schedule, (ii) identify the most responsive patient cohort(s), and (iii) evaluate the efficacy of ADCs as single agents or the need for combination strategies. The clinical development of GO and InO provides a paradigmatic example: the initial toxicity of these compounds was mitigated through dose and schedule optimization, as well as through combination with chemotherapy or bsAbs, thereby counterbalancing the narrow therapeutic window associated with their shared payload [78,99]. Nevertheless, payload-related toxicity remains a major limitation of ADC therapy. For instance, MMAE-associated peripheral neuropathy and MMAF-related corneal toxicity may necessitate dose reductions or even treatment discontinuation. Similarly, highly potent payloads, such as PBD dimers in loncastuximab tesirine, impose drug design limits, requiring low DARs and therefore narrowing the therapeutic window. Importantly, linker stability and its PK properties, which are discussed below in a dedicated section, directly affect payload release and downstream effects. Accordingly, ADC efficacy depends not only on payload choice, but on its optimal pairing with the linker, as the two act synergistically to maximize therapeutic activity.

Clinical experience with ADCs has also shown that antigen expression alone is insufficient to predict efficacy. Although ADCs require a minimal antigen density to exert adequate therapeutic effects, stability over time critically influences treatment outcomes. Antigens, such as CD22 and CD79b, exhibit a rapid and efficient internalization following ADC binding, resulting in an effective payload delivery and marked clinical activity, as in the case of InO and Pola-V. Similarly, the relatively stable expression and internalization of CD33 may partially explain the durable responses achieved with BV. In contrast, BCMA expression is more dynamic, with documented antigen downregulation and shedding under therapeutic pressure, contributing to antigen escape and limiting the durability of responses to BCMA-targeted ADCs, such as belantamab mafodotin [2,100].

A final critical point that needs to be considered is the identification of patient subgroups that would benefit most from ADC treatment. To this end, it is essential to genetically and molecularly profile patient cohorts to define their common disease biological features, as exemplified by GO administration in patients with CBF AML. This “molecularly-driven approach” allows for the use of ADCs in clinical subgroups in which efficacy is maximized, while minimizing or avoiding the risk of exposing patients to toxicity when therapeutic benefit would be limited.

Collectively, these data highlight how antigen expression, internalization, and recycling directly impact on clinical outcomes and potentially in resistance phenomena, underscoring the importance of integrating antigen biology with patient selection, treatment schedule, and ADC design. Moreover, the different therapeutic responses observed indicate that the use of ADCs in clinics must follow a “personalized” approach.

## 5. ADCs’ Current Limitations

Although ADCs have demonstrated significant therapeutic activities and currently represent a rapidly growing class of anticancer agents, several challenges still limit their clinical utility.

(i) Less than 1% of the total dose of intravenously administered ADCs reaches tumor cells, whereas approximately one third of the injected dose remains in circulation and up to 15% distributes to the liver, with additional uptake in the spleen, kidneys, and adipose tissue [101,102,103]. This issue is mainly relevant for those ADCs targeting antigens that are not exclusively tumor-associated antigens, thus presenting a broader expression, including normal cells [102]. Defining their toxicity mechanisms is therefore crucial to design safer and more selective compounds.
(ii) Cancers are usually characterized by intra- and inter-tumoral heterogeneity. On the one side, intratumoral heterogeneity is the result of changes over time in antigen expression and density, or the presence of cells with a more aggressive phenotype. These features can determine suboptimal drug responses or the emergence of ADC-resistant clones during treatment, ultimately resulting in disease relapse. On the other hand, the same type of neoplasia can be genetically and/or molecularly different across patients, underscoring the need to optimize therapeutic strategies based on disease profiling and the implementation of patient-tailored treatments [104].
(iii) The conjugated payload and linker used in ADC design, independently of the target antigen, contribute to the dose-limiting toxicity (DLT) and maximum tolerated dose (MTD). Data from clinical ADC development programs have shown that compounds sharing the same linker–payload exhibit highly similar toxicity profiles, DLTs, and MTDs, largely independent of the target antigen or its expression in normal tissues [102,105]. For instance, vc-MMAE-based ADCs consistently induce bone marrow suppression, sepsis, and peripheral neuropathy, a pattern observed across multiple clinical candidates and approved agents, such as Pola-V [28,106]. ADCs sharing the same payload may show a similar toxicity profile. Specifically, MMAE-containing ADCs are commonly associated with neutropenia and neuropathy, DM1-based ADCs with thrombocytopenia and hepatotoxicity, MMAF- or DM4-containing ADCs with ocular toxicity, and PBD dimer ADCs with bone marrow suppression, vascular leak syndrome, and hepatic and renal adverse events [105,107]. Illustrative clinical examples further highlight this principle. Trastuzumab-based ADCs armed with distinct payloads exhibit non-overlapping toxicities, with trastuzumab emtansine (DM1 payload) primarily causing thrombocytopenia and hepatotoxicity, whereas trastuzumab deruxtecan is associated with interstitial lung disease and enhanced myelosuppression [108]. Similarly, BCMA-targeted ADCs show divergent DLTs depending on the payload, with MMAF-based belantamab mafodotin characterized by ocular toxicity, while alternative warheads confer distinct systemic adverse events [93]. CD33- and CD22-targeted ADCs conjugated to calicheamicin, such as GO and InO, are consistently associated with hepatotoxicity and sinusoidal obstruction syndrome [24,109,110]. Finally, toxicity analyses stratified by ADC payload and linker type showed higher rates of grade ≥3 adverse events (meaning severe or medically significant, but not immediately life-threatening events, according to CTCAE criteria [111]) among ADCs with cleavable linkers compared to those with non-cleavable ones (7.9% vs. 2.2%, respectively) [112].

In addition to the limitations discussed above, the development of acquired resistance during treatment represents a major drawback to ADC efficacy [113]. This clinical issue may arise at different levels:

(i) The downregulation of the surface target antigen during therapy, which ultimately leads to treatment refractoriness. While this phenomenon has been reported for treatment with anti-BCMA ADCs (e.g., belantamab mafodotin), it appears to be less relevant and frequent for other ADCs, including the anti-CD33 GO and the anti-CD30 BV [114,115,116].
(ii) Antigen escape occurring because of genetic mutations or structural alterations of the target antigen, as described for anti-CD22 therapy with InO [114].
(iii) Alteration in proteins involved in endocytic trafficking or lysosomal dysfunctions, such as changes in lysosomal pH or defects in transmembrane transport, which are critical determinants for payload release [113].
(iv) Payload target or target-related protein alterations, as reported for anti-CD22 ADC InO, where mutations in key genes or pathways involved in DNA repair lead to reduced sensitivity to calicheamicin [117].
(v) The overexpression of ATP-binding cassette transporters on the tumor cell surface. This mechanism has been described in resistance to both GO and BV, as these efflux pumps promote the extrusion of the cytotoxic payload from tumor cells [115,116].
(vi) The dysregulation of apoptotic pathways, as in the case of GO therapy, where the overexpression of the anti-apoptotic proteins BCL-2 and BCL-xL has been shown to contribute to resistance [116].

To overcome these limitations, multiple strategies have been explored to develop increasingly effective and selective compounds, ultimately aiming to minimize the incidence of off-target or unexpected toxicities and to avoid resistance.

## 6. Conclusions and Future Directions

The evolution of ADCs has been driven by the iterative optimization of each structural component—antibody, linker, and cytotoxic payload—leading to next-generation compounds with improved physical–chemical properties, efficacy, and safety profiles.

Starting from the antibody, recent developments in antibody engineering have centered on optimizing epitope selection, binding affinity, and surface charge to enhance therapeutic specificity while minimizing off-tumor toxicity. Targeting epitopes that promote rapid receptor-mediated internalization or employing bsAbs improve ADC uptake and potency, even for tumors characterized by low antigen density [5,118]. The fine tuning of binding affinity remains critical to balance sufficient target engagement with reduction of on-target/off-tumor adverse effects. Furthermore, controlled charge alterations of the antibody structure have been shown to reduce target-independent toxicities and related adverse events [119].

Humanized or fully human IgG backbones remain the preferred scaffolds for clinical ADC development. IgG1 is now favored over IgG4 for its longer serum half-life, structural robustness, and capacity for FcγR engagement, while maintaining limited immunogenicity [2,4]. Most ADCs retain the N297-linked Fc glycan, which supports FcγR binding but can promote nonspecific hepatic uptake via mannose receptors. To mitigate hepatotoxicity, a-glycosylated antibodies are currently being explored, and the conjugation of small-molecule payloads to the CH2 domain of the antibody has been reported to restore structural stability [120].

In parallel, next-generation designs, such as bispecific ADCs (bsADCs) that simultaneously target multiple tumor antigens or two distinct epitopes on the same antigen (biparatopic design) pave the way for more selective and highly effective compounds [120,121]. Indeed, bsADCs can induce receptor clustering and accelerate internalization, thereby enhancing lysosomal processing and increasing payload release compared with conventional ADCs [121]. By engaging two targets or epitopes, bsADCs have the potential to bypass key limitations of conventional ADCs, resulting in improved tumor selectivity while limiting the off-tumor on-target mechanism, and mitigating the effects of antigen heterogeneity [120,122,123]. This strategy is exemplified by biparatopic ADCs targeting HER2, where epitope selection has been critical to modulating receptor trafficking, leading to enhanced internalization and improved cytotoxic activity [124]. A further example of the improved efficacy of bsADCs is represented by HER2×PRLR bsADCs that showed a marked enhanced activity over monospecific HER2 ADCs in double-positive cells, with an approximately 100-fold reduction in EC_50_, driven by PRLR-mediated internalization and lysosomal trafficking [125,126]. Additional bispecific designs, such as ADCs targeting HER2 and the lysosomal membrane protein CD63, further support the concept that redirecting ADCs toward lysosomal compartments can enhance payload delivery and antitumor activity [127]. Despite these advancements, bsADC development remains largely limited to solid tumors, with no constructs, in advanced clinical stages, capable of targeting onco-hematological antigens.

Although the ideal ADC target would be exclusively expressed on malignant cells, many clinically validated antigens, such as CD19, are also found in normal counterparts, leading to target-mediated toxicities. Novel strategies to enhance tumor selectivity thus include the use of antibodies recognizing TAAs [121]. In line with this view, the application of transcriptomic and proteomic analyses to compare normal versus cancer cells is a driving force in the identification of such antigens, opening up opportunities for more selective compounds. An alternative strategy is represented by the use of conditionally activated constructs such as probody–drug conjugates (PDCs) [128]. Probody technology represents an innovative strategy to address the challenge of on-target/off-tumor toxicity by exploiting the dysregulated protease activity typical of the TME. PDCs remain inert in systemic circulation and are selectively activated upon proteolytic cleavage within the TME, where elevated protease activity or acidic pH can unmask antigen-binding sites, resulting in localized payload release. This approach allows side effects associated with broadly expressed antigens to be reduced while improving tumor selectivity and efficacy [120]. CX-2029, an anti-CD71 MMAE-conjugated PDC, demonstrates the clinical potential of this approach. Preclinical studies in non-human primates showed a 10-fold increase in tolerable dose compared with the unmasked ADC, allowing efficient payload delivery while minimizing systemic toxicity [129,130]. The preclinical efficacy of CX-2029 in multiple patient-derived xenograft (PDX) models, including DLBCL, supported its advancement into a phase I-II first-in-human clinical trial (PROCLAIM-CX-2029) [130,131]. Alternative masking strategies, such as heterodimeric coiled-coil domains, offer steric or ion-dependent conditional masking and have demonstrated broad applicability across hematologic targets such as CD19 and CD20 in preclinical models [120,132].

A second area of innovation in ADC performance is represented by the linker. Far from being an inert connector, it profoundly influences the balance between systemic stability and efficient payload release. Advances in linker chemistry have markedly reduced premature systemic deconjugation, improved PK, and enhanced therapeutic efficacy [5]. One key approach involves the reduction of compound hydrophobicity through linker modifications. Lowering ADC hydrophobicity has been shown to enhance both PK and antitumor activity, partly by mitigating micropinocytosis-mediated off-target uptake and nonspecific tissue distribution [133]. Enhanced linker hydrophilicity may also modulate ADCs’ toxicity profile by influencing the bystander effect, potentially through the reduced efflux of payload metabolites via MDR1 transporters, although this outcome appears to be construct-dependent [134].

Another strategy focuses on increasing the DAR without compromising molecular stability. Conventional ADCs, bearing multiple hydrophobic payloads, often face issues such as aggregation and accelerated clearance. The use of polymer-based linkers, PEGylated chains, or hydrophilic peptide-based linkers can expand drug loading capacity while maintaining solubility and favorable PK properties [133,135]. In addition to hydrophobicity and DAR, linker length and proximal steric hindrance near the conjugation site are structural variables that influence ADC stability. Tuning of these parameters reduces premature payload release by limiting unwanted interactions of the linker–antibody conjugate with serum components and circulating enzymes, thus improving systemic exposure prior to target engagement [136].

Furthermore, innovations in cleavable linker design aim to restrict payload release to the tumor compartment while preventing systemic exposure. Sequentially cleavable systems, such as glucuronidase-sensitive linkers that unmask a secondary cathepsin site, ensure that both enzymatic steps occur exclusively within lysosomes. These tandem-cleavage linkers have demonstrated improved stability and tolerability in preclinical studies, highlighting their promise for broadening the therapeutic window of next-generation ADCs [137].

Payload selection represents a final area of innovation in ADC design. Effective drugs must display adequate mechanism of cytotoxicity, in addition to plasma stability, and chemical compatibility with conjugation processes, while preserving solubility and enabling efficient intracellular release. These requirements have driven the development of novel cytotoxic agents, as well as the design of dual-payload constructs and prodrug systems. One opportunity of innovation involves the design of hydrophilic cytotoxic payloads, which allows for high-DAR ADCs without compromising molecular stability or PK. The glycosylated auristatin β-D-glucuronide (MMAU) well exemplifies this strategy: it remains inert in its masked form and regains potent intracellular cytotoxic activity following lysosomal deglycosylation, while exhibiting minimal bystander effects due to its limited membrane permeability [138].

Dual-payload ADCs represent another frontier in payload development. These constructs integrate two mechanistically distinct cytotoxic agents within a single molecule, enhancing tumoricidal efficacy and mitigating resistance [139]. An example is provided by KH815, a novel TROP2-directed dual-payload ADC carrying a TOP1 inhibitor and an RNA polymerase II inhibitor, which has shown potent antitumor activity in preclinical models and a favorable safety profile [140].

Prodrug-based payloads have been designed to minimize off-tumor toxicity by exploiting tumor-specific features, such as acidic pH, hypoxia, hyper-sialylation, and elevated protease activity [141]. In these systems, highly potent or hydrophobic cytotoxins are transiently masked becoming activated only within the TME, where enzymatic or non-enzymatic cleavage restores their cytotoxic function [142]. In addition to the above discussed payloads already approved for ADC, other compounds with different mechanisms of action have entered this scenario, currently undergoing various stages of clinical and preclinical investigation. They are mainly represented by transcription, TOP2, BCL-xL, and tyrosine kinase (TK) inhibitors [7]. Among transcription inhibitors, antibody-targeted amanitin conjugates (ATACs) and histone deacetylase inhibitors (HDACi) represent a notable example of advanced payloads. By blocking RNA polymerase II, amanitin exerts potent cytotoxicity even in quiescent cells, addressing a key limitation of classical antimitotic payloads. Early clinical candidates, such as anti-BCMA or anti-CD37 ADCs carrying amanitin derivatives, have shown promising preclinical efficacy and are now being evaluated [143,144,145].

More recently, not only potent cytotoxic agents, but also immune modulators, protein degradation inducers, and metabolic inhibitors have been considered as potential ADC payloads. Besides emerging as therapeutic tools in the oncology field, they can be promising drugs in the setting of chronic inflammatory and autoimmune diseases [5,7]. For instance, immune-stimulating antibody conjugates (ISACs) combine targeted delivery with immune activation, employing antibodies conjugated to immune agonists (mainly Toll-like receptors—TLRs—and stimulator of interferon genes—STING) to promote local immune responses within the TME. Early clinical examples, including anti-HER2 antibodies conjugated to TLR7/8 agonists, have demonstrated encouraging preclinical efficacy and tolerability [7]. PROTACs (Proteolysis Targeting Chimeric Molecules) also represent a novel class of bifunctional agents, inducing proteasomal degradation of targets recruiting E3 ligases [146]. Integration into antibody-based constructs has led to the development of Degrader–Antibody Conjugates (DACs) or Antibody neoDegrader Conjugates (AnDCs), which exploit antibody-mediated delivery to overcome the poor cell permeability of PROTACs. Different strategies are also represented by ADCs delivering NAMPT inhibitors, showing selective NAD depletion and potent in vivo antitumor activity revealing metabolic interference as a promising therapeutic avenue [147]. Similarly, kinesin spindle protein (KSP) inhibitors specifically target proliferating cells, potentially avoiding undesired toxicities.

Overall, this review provides an overview of the current landscape of ADC development in hematological malignancies. Six ADCs have already been approved by the FDA and EMA, representing a substantial portion of the therapeutic arsenal available to treat these patients (Table 1). These agents have often reshaped the treatment paradigm, and have significantly improved prognosis, while expanding therapeutic options. Besides the approved compounds, the interest in this field is witnessed by the overall number of ADCs that have been included in preclinical and clinical investigations for hematological malignancies over the last decade (Table 2 and Table 3).

Although ADCs currently constitute an important component of targeted therapies, the field is rapidly evolving, driven by an increasingly refined understanding of the mechanisms underlying cancer biology and ADC design and engineering. Modifications to individual drug components allow for the development of compounds showing greater specificity while minimizing unwanted toxicities, finally broadening the spectrum of patients that may benefit from these treatments.

Accordingly, we have discussed forthcoming therapeutic innovations that will introduce into the clinics increasingly sophisticated ADCs, further expanding treatment possibilities for patients whose management remains challenging, particularly in fragile cohorts and in the context of frequent disease relapses that characterize the natural history of hematological malignancies.

## Figures and Tables

**Table 1:** Food and Drug Administration (FDA)- and European Medicine Agency (EMA)-approved antibody–drug conjugates for hematological malignancies and their main related toxicities. AML: acute myeloid leukemia; APL: acute promyelocytic leukemia; TN: treatment-naïve; cHL: classical Hodgkin lymphoma; ASCT: autologous stem cell transplantation; sALCL: systemic anaplastic large-cell lymphoma; CHP: cyclophosphamide, doxorubicin, prednisone; R/R: relapsed/refractory; CTCL: cutaneous T-cell lymphoma; DLBCL: diffuse large-B-cell lymphoma; LBCL: large-B-cell-lymphoma; R-CHP: rituximab-CHP; HGBL: high-grade B-lymphoma; B-ALL: B-lineage acute lymphoblastic leukemia; Ph+: Philadelphia positive; TKI: tyrosine kinase inhibitor; RRMM: relapsed or refractory multiple myeloma; BCMA: B-cell maturation antigen; CL: cleavable; Non-Cl: non-cleavable. * (EMA).

ADC Name	Target	Payload	Linker	DAR	mAb	Approved Indications	Main Related Toxicities	Ref.
Gemtuzumab ozogamicin	CD33	N-acetyl-gamma-calicheamicin	Cl	2.5	IgG4k	CD33+ untreated AML in patients > 15 years, except APL, in combination with daunorubicin and cytarabine (EMA) CD33+ untreated AML in adult patients; CD33+ R/R AML in patients > 2 years. GO can be used in combination with daunorubicin and cytarabine and as monotherapy (FDA)	HVOD/SOS Myelosuppression (thrombocytopenia, neutropenia) Infections	[16,17,18]
Brentuximab vedotin	CD30	Monomethyl auristatin E (MMAE)	Cl	4	IgG1	CD30+ TN cHL stage III/IV in association with AVD (EMA/FDA) CD30+ TN cHL stage IIB/III/IV in association with etoposide, cyclophosphamide, doxorubicin, dacarbazine and dexamethasone (EMA) CD30+ TN high risk cHL in pediatric patients in association with doxorubicin, vincristine, etoposide, prednisone and cyclophosphamide (FDA) CD30+ high risk cHL maintenance after ASCT (EMA/FDA) CD30+ cHL after ASCT failure or after failure of at least two prior treatment lines (EMA/FDA) TN sALCL in association to CHP (EMA/FDA) R/R sALCL (EMA/FDA) CD30+ R/R CTCL (EMA/FDA) R/R LBCL after two or more lines of systemic therapy not eligible for ASCT or CAR-T therapy in combination with lenalidomide and rituximab (FDA)	Peripheral sensory neuropathy Myelosuppression (neutropenia) GI symptoms (nausea, diarrhea, decreased appetite) Elevated liver enzymes Pruritus	[19,20,21,22]
Inotuzumab ozogamicin	CD22	N-acetyl-gamma-calicheamicin	Cl	6	IgG4k	R/R CD22+ adult B-ALL (EMA); R/R CD22+ pediatric and adult B-ALL (FDA) B-ALL Ph+ after TKI treatment failure (EMA/FDA)	Myelosuppression (thrombocytopenia, neutropenia) Elevated liver enzymes GI symptoms (nausea and vomiting) HVOD/SOS	[23,24,25,26]
Conatumumab vedotin	CD79b	Monomethyl auristatin E (MMAE)	Cl	3.5	IgG1	R/R DLBCL in combination with BR (EMA/FDA) Previously untreated DLBCL in association to R-CHP (EMA/FDA)	Peripheral sensory neuropathy Myelosuppression (neutropenia) GI symptoms (nausea and vomiting, diarrhea, decreased appetite)	[27,28,29]
Loncastuximab tesirine	CD19	Pyrrolobenzodiazepine (PBD)	Cl	2	IgG1	R/R DLBCL and HGBL monotherapy after two lines of systemic therapy (EMA/FDA)	Myelosuppression (thrombocytopenia, neutropenia, anemia) Hepatotoxicity Peripheral edema GI symptoms (nausea and vomiting, diarrhea, decreased appetite) Skin-related toxicity (rash)	[30,31,32]
Belantamab mafodotin *	BCMA	Monomethyl auristatin F (MMAF)	Non-Cl	2.5	IgG1	In combination with bortezomib and desametasone (BVd) in RRMM patients (EMA) In combination with pomalidomide and desametasone (Pd) in RRMM patients after at least one lenalidomide-based therapy (EMA)	Ocular toxicity (mainly corneal epithelium alterations) Myelosuppression (thrombocytopenia, anemia)	[33]

**Table 2:** Overview of ADCs under preclinical investigation for hematological malignancies in the last decade. B-ALL: B-lineage acute lymphoblastic leukemia; DLBCL: diffuse large-B-cell lymphoma; NHL: non-Hodgkin lymphoma; RT: Richter transformation; AML: acute myeloid leukemia; CTCL: cutaneous T-cell lymphoma; MM: multiple myeloma; ALL: acute lymphoblastic leukemia; CLL: chronic lymphocytic leukemia; HL: Hodgkin lymphoma; Cl: cleavable; Non-Cl: non-cleavable. NA: not available.

Compound Name	Target	Payload	Linker	Experimental Model	References
huB4-DGN462	CD19	DGN462 (IGN)	Cl	B-ALL and B-lymphoma cell lines, DLBCL xenograft models	[148]
Anti–CD22-(LC:K149C)-SN3624	CD22	SN3624 (seco-CBI-dimer)	Non-Cl	NHL xenograft models	[149]
CAT-02-106	CD22	Maytansine	Non-Cl	NHL xenograft models	[150]
PF-08046032	CD25	MMAE	NA	Lymphoma xenograft models	[151]
SGN-CD30c	CD30	AMDCPT (TOP1 inhibitor)	Cl	Lymphoma preclinical models	[152]
Anti-CD37 Ama1, 2, 3	CD37	Amanitin	Cl	RT xenograft models	[143]
IMGN529 (Naratuximab emtansine)	CD37	DM1 (Maytasinoid)	Non-Cl	AML cell lines, primary AML blasts and xenograft models	[153]
STRO-001	CD74	SC347 (Maytasinoid)	Non-Cl	AML cell lines and xenograft models; CTCL cell lines and xenograft models	[154,155]
STI-8811	BCMA	Duostatin	Cl	MM cell lines and xenograft models	[156]
huXBR1-402-G5-PNU	ROR1	PNU (anthracycline derivative)	NA	ROR1+ leukemia xenograft models	[88]
IMMU-140	HLA-DR	SN38 (TOP1 inhibitor)	NA	AML, ALL, CLL, MM cell lines HL and DLBCL xenograft models	[157]
CD123-CPI-ADC	CD123	CPI	NA	AML xenograft models	[158]

**Table 3:** Overview of ADCs under clinical investigation (active, recruiting or completed) for hematological malignancies in the last decade. AML: acute myeloid leukemia; cHL: classical Hodgkin lymphoma; NHL: non-Hodgkin lymphoma; R/R: relapsed/refractory; DLBCL: diffuse large-B-cell lymphoma; MCL: mantle cell lymphoma; MZL: marginal zone lymphoma; B-ALL: B-lineage acute lymphoblastic leukemia; R/R MM: relapsed or refractory multiple myeloma; R/R BPDCN: Relapsed/Refractory Blastic Plasmacytoid Dendritic Cell Neoplasm; CMML: chronic myelomonocytic leukemia; RT: Richter transformation; FL: follicular lymphoma; CLL: chronic lymphocytic leukemia; MDS: myelodysplastic syndrome; R-CHP: rituximab-CHP; R-CHOP: rituximab-CHOP; SOC: standard of care; Cl: cleavable; Non-Cl: non-cleavable. The indicated stage of clinical advancement is based on information available on ClinicalTrials.gov updated in December 2025.

Compound Name	Target	Payload	Linker	Clinical Field	Clinical Phase	Trial ID	Status	References
ABBV-319	CD19	GRM (glucocorticoid receptor modulator)	Cl	R/R DLBCL, FL, CLL	I	NCT05512390	Recruiting	[159]
TRPH-222	CD22	Maytansine	Non-Cl	R/R B-cell malignancies (DLBCL, FL, MCL, MZL)	I	NCT03682796	Completed	[160]
ADCT-602	CD22	SG3249 (PBD)	Cl	R/R B-ALL	I/II	NCT03698552	Recruiting	[161]
ADCT-301 (*Camidanlumab tesirine*)	CD25	SG3199 (PBD)	Cl	R/R NHL and cHL	I	NCT02432235	Completed	[162,163,164]
				R/R cHL	II	NCT04052997	Completed	
F0002-ADC	CD30	DM1 (Maytasinoid)	Non-Cl	R/R CD30+ hematological malignancies	I	NCT03894150 (China)	Completed	[165]
BL-M11D1	CD33	Ed-04 (TOP1 inhibitor)	Cl	R/R AML	I	NCT05924750	Recruiting	[99]
IMGN529 (*Naratuximab emtansine*) Debio 1562M	CD37	DM1 (Maytasinoid)	Non-Cl	R/R B-cell malignancies (DLBCL, FL, MCL, MZL)	I	NCT01534715	Completed	[166,167,168]
				R/R DLBCL in combination with rituximab	II	NCT02564744	Completed	
IMGN632 (*Pivekimab Sunirine*)	CD123	sFGN849 (IGN)	Cl	Untreated or R/R BPDCN	I/II	NCT03386513	Active	[169,170]
				AML monotherapy or in combination with venetoclax and/or azacitidine	I/II	NCT04086264	Active	
				Newly diagnosed adverse risk AML and other high-grade myeloid neoplasms in combination with FLAG-Ida	I	NCT06034470	Recruiting	
AZD9829	CD123	AZ14170132 (TOP1 inhibitor)	NA	CD123+ hematological malignancies	I/II	NCT06179511	Recruiting	[171]
S227928	CD74	S64315 (MCL-1 inhibitor)	Cl	R/R AML, MDS/AML, CMML monotherapy or in combination with venetoclax	I	NCT06563804	Recruiting	[172]
STRO-001	CD74	SC347 (Maytasinoid)	Non-Cl	Advanced B-cell malignancies (MM and NHL)	I	NCT03424603	Completed	[173,174]
VLS-101/MK-2140 (*Zilovertamab vedotin*)	ROR1	MMAE (Auristatin)	Cl	Pediatric R/R B-ALL, DLBCL, BL	I/II	NCT06395103	Recruiting	[32,175]
				Aggressive and indolent B-cell malignancies (MCL, RT, FL, CLL) monotherapy and in combination	II	NCT05458297	Recruiting	
				R/R DLBCL in combination with SOC (R-GemOx/BR) versus SOC	II/III	NCT05139017	Recruiting	
				Untreated DLBCL in combination with R-CHP versus R-CHOP	III	NCT06717347	Recruiting	
				Untreated DLBCL in combination with R-CHP versus Pola-R-CHP	II	NCT06890884	Recruiting	
CS5001	ROR1	PBD dimer	Cl	Lymphomas as single agent and in combination	I	NCT05279300	Recruiting	[176,177]
STI-6129	CD38	DUO-5.2 (microtubule inhibitor)	NA	R/R MM	I/II	NCT05308225 (US) NCT05565807 (China)	Active	[178]
TAK-573 (*Modakafusp Alfa*)	CD38	IFNα2b	NA	R/R MM	II	NCT03215030	Completed	[179,180,181]
FOR46	CD46	MMAF (Auristatin)	Non-Cl	R/R MM	I	NCT03650491	Completed	[182]
IMGN901 (*Lorvotuzumab mertansine*)	CD56	DM1 (Maytasinoid)	Cl	R/R MM monotherapy	I	NCT00346255	Completed	[183]
				R/R MM in combination with lenalidomide and dexamethasone	I	NCT00991562	Completed	
HDP-101	BCMA	α-amanitin	Cl	R/R MM	I/II	NCT04879043	Recruiting	[145]
BT062 (*Indatuximab ravntansine*)	CD138	DM4 (Maytasinoid)	Cl	R/R MM monotherapy	I/II	NCT01001442 NCT00723359	Completed	[184,185]
				R/R MM in combination with pomalidomide or lenalidomide and dexamethasone	I/II	NCT01638936	Completed	

## Data Availability

No new data were created or analyzed in this study. Data sharing is not applicable to this article.

## References

[1] Colombo R., Tarantino P., Rich J.R., LoRusso P.M., de Vries E.G.E. (2024). The Journey of Antibody-Drug Conjugates: Lessons Learned from 40 Years of Development. Cancer Discov..

[2] Wang R., Hu B., Pan Z., Mo C., Zhao X., Liu G., Hou P., Cui Q., Xu Z., Wang W. (2025). Antibody-Drug Conjugates (ADCs): Current and future biopharmaceuticals. J. Hematol. Oncol..

[3] Shi R., Jia L., Lv Z., Cui J. (2025). Another power of antibody-drug conjugates: Immunomodulatory effect and clinical applications. Front. Immunol..

[4] Yang C., He B., Zhang H., Wang X., Zhang Q., Dai W. (2023). IgG Fc Affinity Ligands and Their Applications in Antibody-Involved Drug Delivery: A Brief Review. Pharmaceutics.

[5] Maecker H., Jonnalagadda V., Bhakta S., Jammalamadaka V., Junutula J.R. (2023). Exploration of the antibody-drug conjugate clinical landscape. mAbs.

[6] Bargh J.D., Isidro-Llobet A., Parker J.S., Spring D.R. (2019). Cleavable linkers in antibody-drug conjugates. Chem. Soc. Rev..

[7] Conilh L., Sadilkova L., Viricel W., Dumontet C. (2023). Payload diversification: A key step in the development of antibody–drug conjugates. J. Hematol. Oncol..

[8] Tsuchikama K., An Z. (2018). Antibody-drug conjugates: Recent advances in conjugation and linker chemistries. Protein Cell.

[9] Dere R.C., Beardsley R.L., Lu D., Lu T., Ku G.H., Man G., Nguyen V., Kaur S. (2023). Integrated summary of immunogenicity of polatuzumab vedotin in patients with relapsed or refractory B-cell non-Hodgkin’s lymphoma. Front. Immunol..

[10] Dornan D., Bennett F., Chen Y., Dennis M., Eaton D., Elkins K., French D., Go M.A., Jack A., Junutula J.R. (2009). Therapeutic potential of an anti-CD79b antibody-drug conjugate, anti-CD79b-vc-MMAE, for the treatment of non-Hodgkin lymphoma. Blood.

[11] Van de Donk N.W., Dhimolea E. (2012). Brentuximab vedotin. mAbs.

[12] Bross P.F., Beitz J., Chen G., Chen X.H., Duffy E., Kieffer L., Roy S., Sridhara R., Rahman A., Williams G. (2001). Approval summary: Gemtuzumab ozogamicin in relapsed acute myeloid leukemia. Clin. Cancer Res..

[13] Rubinstein J.D., O’Brien M.M. (2023). Inotuzumab ozogamicin in B-cell precursor acute lymphoblastic leukemia: Efficacy, toxicity, and practical considerations. Front. Immunol..

[14] Senter P.D. (2009). Potent antibody drug conjugates for cancer therapy. Curr. Opin. Chem. Biol..

[15] Ferron-Brady G., Rathi C., Collins J., Struemper H., Opalinska J., Visser S., Jewell R.C. (2021). Exposure-Response Analyses for Therapeutic Dose Selection of Belantamab Mafodotin in Patients With Relapsed/Refractory Multiple Myeloma. Clin. Pharmacol. Ther..

[16] Collados-Ros A., Muro M., Legaz I. (2024). Gemtuzumab Ozogamicin in Acute Myeloid Leukemia: Efficacy, Toxicity, and Resistance Mechanisms-A Systematic Review. Biomedicines.

[17] Cortes J.E., de Lima M., Dombret H., Estey E.H., Giralt S.A., Montesinos P., Rollig C., Venditti A., Wang E.S. (2020). Prevention, recognition, and management of adverse events associated with gemtuzumab ozogamicin use in acute myeloid leukemia. J. Hematol. Oncol..

[18] Montesinos P., Kota V., Brandwein J., Bousset P., Benner R.J., Vandendries E., Chen Y., McMullin M.F. (2023). A phase IV study evaluating QT interval, pharmacokinetics, and safety following fractionated dosing of gemtuzumab ozogamicin in patients with relapsed/refractory CD33-positive acute myeloid leukemia. Cancer Chemother. Pharmacol..

[19] Younes A., Gopal A.K., Smith S.E., Ansell S.M., Rosenblatt J.D., Savage K.J., Ramchandren R., Bartlett N.L., Cheson B.D., de Vos S. (2012). Results of a pivotal phase II study of brentuximab vedotin for patients with relapsed or refractory Hodgkin’s lymphoma. J. Clin. Oncol..

[20] Prince H.M., Kim Y.H., Horwitz S.M., Dummer R., Scarisbrick J., Quaglino P., Zinzani P.L., Wolter P., Sanches J.A., Ortiz-Romero P.L. (2017). Brentuximab vedotin or physician’s choice in CD30-positive cutaneous T-cell lymphoma (ALCANZA): An international, open-label, randomised, phase 3, multicentre trial. Lancet.

[21] Chen R., Palmer J.M., Martin P., Tsai N., Kim Y., Chen B.T., Popplewell L., Siddiqi T., Thomas S.H., Mott M. (2015). Results of a Multicenter Phase II Trial of Brentuximab Vedotin as Second-Line Therapy before Autologous Transplantation in Relapsed/Refractory Hodgkin Lymphoma. Biol. Blood Marrow Transplant..

[22] Forero-Torres A., Holkova B., Goldschmidt J., Chen R., Olsen G., Boccia R.V., Bordoni R.E., Friedberg J.W., Sharman J.P., Palanca-Wessels M.C. (2015). Phase 2 study of frontline brentuximab vedotin monotherapy in Hodgkin lymphoma patients aged 60 years and older. Blood.

[23] DeAngelo D.J., Stock W., Stein A.S., Shustov A., Liedtke M., Schiffer C.A., Vandendries E., Liau K., Ananthakrishnan R., Boni J. (2017). Inotuzumab ozogamicin in adults with relapsed or refractory CD22-positive acute lymphoblastic leukemia: A phase 1/2 study. Blood Adv..

[24] Kantarjian H.M., DeAngelo D.J., Advani A.S., Stelljes M., Kebriaei P., Cassaday R.D., Merchant A.A., Fujishima N., Uchida T., Calbacho M. (2017). Hepatic adverse event profile of inotuzumab ozogamicin in adult patients with relapsed or refractory acute lymphoblastic leukaemia: Results from the open-label, randomised, phase 3 INO-VATE study. Lancet Haematol..

[25] Kantarjian H.M., DeAngelo D.J., Stelljes M., Martinelli G., Liedtke M., Stock W., Gokbuget N., O’Brien S., Wang K., Wang T. (2016). Inotuzumab Ozogamicin versus Standard Therapy for Acute Lymphoblastic Leukemia. N. Engl. J. Med..

[26] Wynne J., Wright D., Stock W. (2019). Inotuzumab: From preclinical development to success in B-cell acute lymphoblastic leukemia. Blood Adv..

[27] Deeks E.D. (2019). Polatuzumab Vedotin: First Global Approval. Drugs.

[28] Palanca-Wessels M.C., Czuczman M., Salles G., Assouline S., Sehn L.H., Flinn I., Patel M.R., Sangha R., Hagenbeek A., Advani R. (2015). Safety and activity of the anti-CD79B antibody-drug conjugate polatuzumab vedotin in relapsed or refractory B-cell non-Hodgkin lymphoma and chronic lymphocytic leukaemia: A phase 1 study. Lancet Oncol..

[29] Tilly H., Morschhauser F., Bartlett N.L., Mehta A., Salles G., Haioun C., Munoz J., Chen A.I., Kolibaba K., Lu D. (2019). Polatuzumab vedotin in combination with immunochemotherapy in patients with previously untreated diffuse large B-cell lymphoma: An open-label, non-randomised, phase 1b-2 study. Lancet Oncol..

[30] Kahl B.S., Hamadani M., Radford J., Carlo-Stella C., Caimi P., Reid E., Feingold J.M., Ardeshna K.M., Solh M., Heffner L.T. (2019). A Phase I Study of ADCT-402 (Loncastuximab Tesirine), a Novel Pyrrolobenzodiazepine-Based Antibody-Drug Conjugate, in Relapsed/Refractory B-Cell Non-Hodgkin Lymphoma. Clin. Cancer Res..

[31] Hamadani M., Radford J., Carlo-Stella C., Caimi P.F., Reid E., O’Connor O.A., Feingold J.M., Ardeshna K.M., Townsend W., Solh M. (2021). Final results of a phase 1 study of loncastuximab tesirine in relapsed/refractory B-cell non-Hodgkin lymphoma. Blood.

[32] Caimi P.F., Ai W.Z., Alderuccio J.P., Ardeshna K.M., Hamadani M., Hess B., Kahl B.S., Radford J., Solh M., Stathis A. (2024). Loncastuximab tesirine in relapsed/refractory diffuse large B-cell lymphoma: Long-term efficacy and safety from the phase II LOTIS-2 study. Haematologica.

[33] Lu R., Morphey A., Diaz F., Chen J., Razmandi A., Richards T. (2023). Management of Ocular Toxicity in Patients Receiving Belantamab Mafodotin. J. Adv. Pract. Oncol..

[34] Ricart A.D. (2011). Antibody-Drug Conjugates of Calicheamicin Derivative: Gemtuzumab Ozogamicin and Inotuzumab Ozogamicin. Clin. Cancer Res..

[35] Lee A. (2021). Loncastuximab Tesirine: First Approval. Drugs.

[36] Petersdorf S.H., Kopecky K.J., Slovak M., Willman C., Nevill T., Brandwein J., Larson R.A., Erba H.P., Stiff P.J., Stuart R.K. (2013). A phase 3 study of gemtuzumab ozogamicin during induction and postconsolidation therapy in younger patients with acute myeloid leukemia. Blood.

[37] Amadori S., Suciu S., Stasi R., Salih H.R., Selleslag D., Muus P., De Fabritiis P., Venditti A., Ho A.D., Lubbert M. (2013). Sequential combination of gemtuzumab ozogamicin and standard chemotherapy in older patients with newly diagnosed acute myeloid leukemia: Results of a randomized phase III trial by the EORTC and GIMEMA consortium (AML-17). J. Clin. Oncol..

[38] Burnett A.K., Hills R.K., Milligan D., Kjeldsen L., Kell J., Russell N.H., Yin J.A., Hunter A., Goldstone A.H., Wheatley K. (2011). Identification of patients with acute myeloblastic leukemia who benefit from the addition of gemtuzumab ozogamicin: Results of the MRC AML15 trial. J. Clin. Oncol..

[39] Burnett A.K., Russell N.H., Hills R.K., Kell J., Freeman S., Kjeldsen L., Hunter A.E., Yin J., Craddock C.F., Dufva I.H. (2012). Addition of gemtuzumab ozogamicin to induction chemotherapy improves survival in older patients with acute myeloid leukemia. J. Clin. Oncol..

[40] Borthakur G., Cortes J.E., Estey E.E., Jabbour E., Faderl S., O’Brien S., Garcia-Manero G., Kadia T.M., Wang X., Patel K. (2014). Gemtuzumab ozogamicin with fludarabine, cytarabine, and granulocyte colony stimulating factor (FLAG-GO) as front-line regimen in patients with core binding factor acute myelogenous leukemia. Am. J. Hematol..

[41] Lambert J., Pautas C., Terre C., Raffoux E., Turlure P., Caillot D., Legrand O., Thomas X., Gardin C., Gogat-Marchant K. (2019). Gemtuzumab ozogamicin for de novo acute myeloid leukemia: Final efficacy and safety updates from the open-label, phase III ALFA-0701 trial. Haematologica.

[42] Hills R.K., Castaigne S., Appelbaum F.R., Delaunay J., Petersdorf S., Othus M., Estey E.H., Dombret H., Chevret S., Ifrah N. (2014). Addition of gemtuzumab ozogamicin to induction chemotherapy in adult patients with acute myeloid leukaemia: A meta-analysis of individual patient data from randomised controlled trials. Lancet Oncol..

[43] Norsworthy K.J., Ko C.W., Lee J.E., Liu J., John C.S., Przepiorka D., Farrell A.T., Pazdur R. (2018). FDA Approval Summary: Mylotarg for Treatment of Patients with Relapsed or Refractory CD33-Positive Acute Myeloid Leukemia. Oncologist.

[44] Jen E.Y., Ko C.W., Lee J.E., Del Valle P.L., Aydanian A., Jewell C., Norsworthy K.J., Przepiorka D., Nie L., Liu J. (2018). FDA Approval: Gemtuzumab Ozogamicin for the Treatment of Adults with Newly Diagnosed CD33-Positive Acute Myeloid Leukemia. Clin. Cancer Res..

[45] Ali S., Dunmore H.M., Karres D., Hay J.L., Salmonsson T., Gisselbrecht C., Sarac S.B., Bjerrum O.W., Hovgaard D., Barbachano Y. (2019). The EMA Review of Mylotarg (Gemtuzumab Ozogamicin) for the Treatment of Acute Myeloid Leukemia. Oncologist.

[46] Dohner H., Wei A.H., Appelbaum F.R., Craddock C., DiNardo C.D., Dombret H., Ebert B.L., Fenaux P., Godley L.A., Hasserjian R.P. (2022). Diagnosis and management of AML in adults: 2022 recommendations from an international expert panel on behalf of the ELN. Blood.

[47] Bradley A.M., Devine M., DeRemer D. (2013). Brentuximab vedotin: An anti-CD30 antibody-drug conjugate. Am. J. Health Syst. Pharm..

[48] Foyil K.V., Bartlett N.L. (2010). Anti-CD30 Antibodies for Hodgkin lymphoma. Curr. Hematol. Malig. Rep..

[49] Prince H.M., Hutchings M., Domingo-Domenech E., Eichenauer D.A., Advani R. (2023). Anti-CD30 antibody-drug conjugate therapy in lymphoma: Current knowledge, remaining controversies, and future perspectives. Ann. Hematol..

[50] Ansell S.M., Radford J., Connors J.M., Dlugosz-Danecka M., Kim W.S., Gallamini A., Ramchandren R., Friedberg J.W., Advani R., Hutchings M. (2022). Overall Survival with Brentuximab Vedotin in Stage III or IV Hodgkin’s Lymphoma. N. Engl. J. Med..

[51] Borchmann P., Ferdinandus J., Schneider G., Moccia A., Greil R., Hertzberg M., Schaub V., Huttmann A., Keil F., Dierlamm J. (2024). Assessing the efficacy and tolerability of PET-guided BrECADD versus eBEACOPP in advanced-stage, classical Hodgkin lymphoma (HD21): A randomised, multicentre, parallel, open-label, phase 3 trial. Lancet.

[52] Castellino S.M., Pei Q., Parsons S.K., Hodgson D., McCarten K., Horton T., Cho S., Wu Y., Punnett A., Dave H. (2022). Brentuximab Vedotin with Chemotherapy in Pediatric High-Risk Hodgkin’s Lymphoma. N. Engl. J. Med..

[53] Evens A.M., Advani R.H., Helenowski I.B., Fanale M., Smith S.M., Jovanovic B.D., Bociek G.R., Klein A.K., Winter J.N., Gordon L.I. (2018). Multicenter Phase II Study of Sequential Brentuximab Vedotin and Doxorubicin, Vinblastine, and Dacarbazine Chemotherapy for Older Patients With Untreated Classical Hodgkin Lymphoma. J. Clin. Oncol..

[54] Younes A., Bartlett N.L., Leonard J.P., Kennedy D.A., Lynch C.M., Sievers E.L., Forero-Torres A. (2010). Brentuximab vedotin (SGN-35) for relapsed CD30-positive lymphomas. N. Engl. J. Med..

[55] Pro B., Advani R., Brice P., Bartlett N.L., Rosenblatt J.D., Illidge T., Matous J., Ramchandren R., Fanale M., Connors J.M. (2012). Brentuximab vedotin (SGN-35) in patients with relapsed or refractory systemic anaplastic large-cell lymphoma: Results of a phase II study. J. Clin. Oncol..

[56] De Claro R.A., McGinn K., Kwitkowski V., Bullock J., Khandelwal A., Habtemariam B., Ouyang Y., Saber H., Lee K., Koti K. (2012). U.S. Food and Drug Administration approval summary: Brentuximab vedotin for the treatment of relapsed Hodgkin lymphoma or relapsed systemic anaplastic large-cell lymphoma. Clin. Cancer Res..

[57] Moskowitz C.H., Nademanee A., Masszi T., Agura E., Holowiecki J., Abidi M.H., Chen A.I., Stiff P., Gianni A.M., Carella A. (2015). Brentuximab vedotin as consolidation therapy after autologous stem-cell transplantation in patients with Hodgkin’s lymphoma at risk of relapse or progression (AETHERA): A randomised, double-blind, placebo-controlled, phase 3 trial. Lancet.

[58] Horwitz S., O’Connor O.A., Pro B., Trumper L., Iyer S., Advani R., Bartlett N.L., Christensen J.H., Morschhauser F., Domingo-Domenech E. (2022). The ECHELON-2 Trial: 5-year results of a randomized, phase III study of brentuximab vedotin with chemotherapy for CD30-positive peripheral T-cell lymphoma. Ann. Oncol..

[59] Domingo-Domenech E., Pro B., Illidge T., Horwitz S., Trumper L., Iyer S., Advani R., Bartlett N.L., Christensen J.H., Kim W.S. (2025). Brentuximab vedotin plus chemotherapy for the treatment of front-line systemic anaplastic large cell lymphoma: Subgroup analysis of the ECHELON-2 study at 5 years’ follow-up. Blood Cancer J..

[60] Horwitz S.M., Scarisbrick J.J., Dummer R., Whittaker S., Duvic M., Kim Y.H., Quaglino P., Zinzani P.L., Bechter O., Eradat H. (2021). Randomized phase 3 ALCANZA study of brentuximab vedotin vs physician’s choice in cutaneous T-cell lymphoma: Final data. Blood Adv..

[61] Bartlett N.L., Hahn U., Kim W.S., Fleury I., Laribi K., Bergua J.M., Bouabdallah K., Forward N., Bijou F., MacDonald D. (2025). Brentuximab Vedotin Combination for Relapsed Diffuse Large B-Cell Lymphoma. J. Clin. Oncol..

[62] Kantarjian H.M., Boissel N., Papayannidis C., Luskin M.R., Stelljes M., Advani A.S., Jabbour E.J., Ribera J.M., Marks D.I. (2024). Inotuzumab ozogamicin in adult acute lymphoblastic leukemia: Development, current status, and future directions. Cancer.

[63] Clark E.A., Giltiay N.V. (2018). CD22: A Regulator of Innate and Adaptive B Cell Responses and Autoimmunity. Front. Immunol..

[64] Dang N.H., Ogura M., Castaigne S., Fayad L.E., Jerkeman M., Radford J., Pezzutto A., Bondarenko I., Stewart D.A., Shnaidman M. (2018). Randomized, phase 3 trial of inotuzumab ozogamicin plus rituximab versus chemotherapy plus rituximab for relapsed/refractory aggressive B-cell non-Hodgkin lymphoma. Br. J. Haematol..

[65] Fayad L., Offner F., Smith M.R., Verhoef G., Johnson P., Kaufman J.L., Rohatiner A., Advani A., Foran J., Hess G. (2013). Safety and clinical activity of a combination therapy comprising two antibody-based targeting agents for the treatment of non-Hodgkin lymphoma: Results of a phase I/II study evaluating the immunoconjugate inotuzumab ozogamicin with rituximab. J. Clin. Oncol..

[66] Goy A., Forero A., Wagner-Johnston N., Christopher Ehmann W., Tsai M., Hatake K., Ananthakrishnan R., Volkert A., Vandendries E., Ogura M. (2016). A phase 2 study of inotuzumab ozogamicin in patients with indolent B-cell non-Hodgkin lymphoma refractory to rituximab alone, rituximab and chemotherapy, or radioimmunotherapy. Br. J. Haematol..

[67] Dijoseph J.F., Dougher M.M., Armellino D.C., Evans D.Y., Damle N.K. (2007). Therapeutic potential of CD22-specific antibody-targeted chemotherapy using inotuzumab ozogamicin (CMC-544) for the treatment of acute lymphoblastic leukemia. Leukemia.

[68] Kantarjian H., Thomas D., Jorgensen J., Jabbour E., Kebriaei P., Rytting M., York S., Ravandi F., Kwari M., Faderl S. (2012). Inotuzumab ozogamicin, an anti-CD22-calecheamicin conjugate, for refractory and relapsed acute lymphocytic leukaemia: A phase 2 study. Lancet Oncol..

[69] Kantarjian H.M., DeAngelo D.J., Stelljes M., Liedtke M., Stock W., Gokbuget N., O’Brien S.M., Jabbour E., Wang T., Liang White J. (2019). Inotuzumab ozogamicin versus standard of care in relapsed or refractory acute lymphoblastic leukemia: Final report and long-term survival follow-up from the randomized, phase 3 INO-VATE study. Cancer.

[70] Jabbour E., Ravandi F., Kebriaei P., Huang X., Short N.J., Thomas D., Sasaki K., Rytting M., Jain N., Konopleva M. (2018). Salvage Chemoimmunotherapy With Inotuzumab Ozogamicin Combined With Mini-Hyper-CVD for Patients With Relapsed or Refractory Philadelphia Chromosome-Negative Acute Lymphoblastic Leukemia: A Phase 2 Clinical Trial. JAMA Oncol..

[71] Kantarjian H., Haddad F.G., Jain N., Sasaki K., Short N.J., Loghavi S., Kanagal-Shamanna R., Jorgensen J., Khouri I., Kebriaei P. (2023). Results of salvage therapy with mini-hyper-CVD and inotuzumab ozogamicin with or without blinatumomab in pre-B acute lymphoblastic leukemia. J. Hematol. Oncol..

[72] Jabbour E.J., Rousselot P., Gokbuget N., Chevallier P., Kantarjian H.M., Stelljes M. (2025). Inotuzumab Ozogamicin as First-Line Therapy in Acute Lymphoblastic Leukemia. Clin. Lymphoma Myeloma Leuk..

[73] Kantarjian H., Ravandi F., Short N.J., Huang X., Jain N., Sasaki K., Daver N., Pemmaraju N., Khoury J.D., Jorgensen J. (2018). Inotuzumab ozogamicin in combination with low-intensity chemotherapy for older patients with Philadelphia chromosome-negative acute lymphoblastic leukaemia: A single-arm, phase 2 study. Lancet Oncol..

[74] Jabbour E., Short N.J., Senapati J., Jain N., Huang X., Daver N., DiNardo C.D., Pemmaraju N., Wierda W., Garcia-Manero G. (2023). Mini-hyper-CVD plus inotuzumab ozogamicin, with or without blinatumomab, in the subgroup of older patients with newly diagnosed Philadelphia chromosome-negative B-cell acute lymphocytic leukaemia: Long-term results of an open-label phase 2 trial. Lancet Haematol..

[75] Chevallier P., Leguay T., Delord M., Salek C., Kim R., Huguet F., Wartiovaara-Kautto U., Raffoux E., Cluzeau T., Balsat M. (2024). Inotuzumab Ozogamicin and Low-Intensity Chemotherapy in Older Patients With Newly Diagnosed CD22(+) Philadelphia Chromosome-Negative B-Cell Precursor Acute Lymphoblastic Leukemia. J. Clin. Oncol..

[76] Stelljes M., Raffel S., Alakel N., Wasch R., Kondakci M., Scholl S., Rank A., Hanel M., Spriewald B., Hanoun M. (2024). Inotuzumab Ozogamicin as Induction Therapy for Patients Older Than 55 Years With Philadelphia Chromosome-Negative B-Precursor ALL. J. Clin. Oncol..

[77] Jain N., Maiti A., Ravandi F., Konopleva M., Daver N., Kadia T., Pemmaraju N., Short N., Kebriaei P., Ning J. (2021). Inotuzumab ozogamicin with bosutinib for relapsed or refractory Philadelphia chromosome positive acute lymphoblastic leukemia or lymphoid blast phase of chronic myeloid leukemia. Am. J. Hematol..

[78] Stock W., Martinelli G., Stelljes M., DeAngelo D.J., Gokbuget N., Advani A.S., O’Brien S., Liedtke M., Merchant A.A., Cassaday R.D. (2021). Efficacy of inotuzumab ozogamicin in patients with Philadelphia chromosome-positive relapsed/refractory acute lymphoblastic leukemia. Cancer.

[79] Lamb Y.N. (2017). Inotuzumab Ozogamicin: First Global Approval. Drugs.

[80] Sehn L.H., Herrera A.F., Flowers C.R., Kamdar M.K., McMillan A., Hertzberg M., Assouline S., Kim T.M., Kim W.S., Ozcan M. (2020). Polatuzumab Vedotin in Relapsed or Refractory Diffuse Large B-Cell Lymphoma. J. Clin. Oncol..

[81] Morschhauser F., Flinn I.W., Advani R., Sehn L.H., Diefenbach C., Kolibaba K., Press O.W., Salles G., Tilly H., Chen A.I. (2019). Polatuzumab vedotin or pinatuzumab vedotin plus rituximab in patients with relapsed or refractory non-Hodgkin lymphoma: Final results from a phase 2 randomised study (ROMULUS). Lancet Haematol..

[82] Pfeifer M., Zheng B., Erdmann T., Koeppen H., McCord R., Grau M., Staiger A., Chai A., Sandmann T., Madle H. (2015). Anti-CD22 and anti-CD79B antibody drug conjugates are active in different molecular diffuse large B-cell lymphoma subtypes. Leukemia.

[83] Sehn L.H., Hertzberg M., Opat S., Herrera A.F., Assouline S., Flowers C.R., Kim T.M., McMillan A., Ozcan M., Safar V. (2022). Polatuzumab vedotin plus bendamustine and rituximab in relapsed/refractory DLBCL: Survival update and new extension cohort data. Blood Adv..

[84] Tilly H., Morschhauser F., Sehn L.H., Friedberg J.W., Trneny M., Sharman J.P., Herbaux C., Burke J.M., Matasar M., Rai S. (2022). Polatuzumab Vedotin in Previously Untreated Diffuse Large B-Cell Lymphoma. N. Engl. J. Med..

[85] Budde L.E., Olszewski A.J., Assouline S., Lossos I.S., Diefenbach C., Kamdar M., Ghosh N., Modi D., Sabry W., Naik S. (2024). Mosunetuzumab with polatuzumab vedotin in relapsed or refractory aggressive large B cell lymphoma: A phase 1b/2 trial. Nat. Med..

[86] Juarez-Salcedo L.M., Nimkar S., Corazon A.M., Dalia S. (2024). Loncastuximab Tesirine in the Treatment of Relapsed or Refractory Diffuse Large B-Cell Lymphoma. Int. J. Mol. Sci..

[87] Li X., Ding Y., Zi M., Sun L., Zhang W., Chen S., Xu Y. (2017). CD19, from bench to bedside. Immunol. Lett..

[88] Caimi P.F., Ai W., Alderuccio J.P., Ardeshna K.M., Hamadani M., Hess B., Kahl B.S., Radford J., Solh M., Stathis A. (2021). Loncastuximab tesirine in relapsed or refractory diffuse large B-cell lymphoma (LOTIS-2): A multicentre, open-label, single-arm, phase 2 trial. Lancet Oncol..

[89] Hamadani M., Spira A., Zhou X., Liao L., Chen L., Radford J., Ai W., Solh M., Ardeshna K.M., Hess B. (2024). Clinical outcomes of older and younger patients treated with loncastuximab tesirine in the LOTIS-2 clinical trial. Blood Adv..

[90] Epperla N., Lucero M., Bailey T., Mirams L., Cheung J., Amet M., Milligan G., Chen L. (2024). Outcomes with loncastuximab tesirine following CAR T-cell therapy in patients with relapsed or refractory diffuse large B-cell lymphoma. Blood Cancer J..

[91] Alderuccio J.P., Alencar A.J., Schatz J.H., Kuker R.A., Pongas G., Reis I.M., Lekakis L.J., Spiegel J.Y., Sandoval-Sus J., Beitinjaneh A. (2025). Loncastuximab tesirine with rituximab in patients with relapsed or refractory follicular lymphoma: A single-centre, single-arm, phase 2 trial. Lancet Haematol..

[92] Shah N., Chari A., Scott E., Mezzi K., Usmani S.Z. (2020). B-cell maturation antigen (BCMA) in multiple myeloma: Rationale for targeting and current therapeutic approaches. Leukemia.

[93] Mukhopadhyay P., Abdullah H.A., Opalinska J.B., Paka P., Richards E., Weisel K., Trudel S., Mateos M.-V., Dimopoulos M.A., Lonial S. (2025). The clinical journey of belantamab mafodotin in relapsed or refractory multiple myeloma: Lessons in drug development. Blood Cancer J..

[94] Baines A.C., Ershler R., Kanapuru B., Xu Q., Shen G., Li L., Ma L., Okusanya O.O., Simpson N.E., Nguyen W. (2022). FDA Approval Summary: Belantamab Mafodotin for Patients with Relapsed or Refractory Multiple Myeloma. Clin. Cancer Res..

[95] Tzogani K., Penttilä K., Lähteenvuo J., Lapveteläinen T., Lopez Anglada L., Prieto C., Garcia-Ochoa B., Enzmann H., Gisselbrecht C., Delgado J. (2021). EMA Review of Belantamab Mafodotin (Blenrep) for the Treatment of Adult Patients with Relapsed/Refractory Multiple Myeloma. Oncologist.

[96] Dimopoulos M.A., Hungria V.T.M., Radinoff A., Delimpasi S., Mikala G., Masszi T., Li J., Capra M., Maiolino A., Pappa V. (2023). Efficacy and safety of single-agent belantamab mafodotin versus pomalidomide plus low-dose dexamethasone in patients with relapsed or refractory multiple myeloma (DREAMM-3): A phase 3, open-label, randomised study. Lancet Haematol..

[97] Dimopoulos M.A., Beksac M., Pour L., Delimpasi S., Vorobyev V., Quach H., Spicka I., Radocha J., Robak P., Kim K. (2024). Belantamab Mafodotin, Pomalidomide, and Dexamethasone in Multiple Myeloma. N. Engl. J. Med..

[98] Hungria V., Robak P., Hus M., Zherebtsova V., Ward C., Ho P.J., Hájek R., Kim K., Grosicki S., Sia H. (2025). Belantamab mafodotin plus bortezomib and dexamethasone in patients with relapsed or refractory multiple myeloma (DREAMM-7): Updated overall survival analysis from a global, randomised, open-label, phase 3 trial. Lancet Oncol..

[99] Song L., Qi J., Wang Z., Li X., Xiao S., Zhu H., Zhu Y., Wang J. (2024). BL-M11D1, a Novel CD33 Antibody-Drug Conjugate (ADC), in Patients with Relapsed/Refractory Acute Myeloid Leukemia: Initial Results from First-in-Human Phase 1 Study. Blood.

[100] Esapa B., Jiang J., Cheung A., Chenoweth A., Thurston D.E., Karagiannis S.N. (2023). Target Antigen Attributes and Their Contributions to Clinically Approved Antibody-Drug Conjugates (ADCs) in Haematopoietic and Solid Cancers. Cancers.

[101] Bensch F., Smeenk M.M., van Es S.C., de Jong J.R., Schröder C.P., Oosting S.F., Lub-de Hooge M.N., Menke-van der Houven van Oordt C.W., Brouwers A.H., Boellaard R. (2018). Comparative biodistribution analysis across four different (89)Zr-monoclonal antibody tracers-The first step towards an imaging warehouse. Theranostics.

[102] Nguyen T.D., Bordeau B.M., Balthasar J.P. (2023). Mechanisms of ADC Toxicity and Strategies to Increase ADC Tolerability. Cancers.

[103] Oude Munnink T.H., Dijkers E.C., Netters S.J., Lub-de Hooge M.N., Brouwers A.H., Haasjes J.G., Schroder C.P., de Vries E.G. (2010). Trastuzumab pharmacokinetics influenced by extent human epidermal growth factor receptor 2-positive tumor load. J. Clin. Oncol..

[104] Drago J.Z., Modi S., Chandarlapaty S. (2021). Unlocking the potential of antibody-drug conjugates for cancer therapy. Nat. Rev. Clin. Oncol..

[105] Masters J.C., Nickens D.J., Xuan D., Shazer R.L., Amantea M. (2018). Clinical toxicity of antibody drug conjugates: A meta-analysis of payloads. Investig. New Drugs.

[106] Saber H., Leighton J.K. (2015). An FDA oncology analysis of antibody-drug conjugates. Regul. Toxicol. Pharmacol..

[107] Zhu Y., Liu K., Wang K., Zhu H. (2023). Treatment-related adverse events of antibody-drug conjugates in clinical trials: A systematic review and meta-analysis. Cancer.

[108] Ma P., Tian H., Shi Q., Liu R., Zhang Y., Qi X., Chen Y. (2023). High risks adverse events associated with trastuzumab emtansine and trastuzumab deruxtecan for the treatment of HER2-positive/mutated malignancies: A pharmacovigilance study based on the FAERS database. Expert Opin. Drug Saf..

[109] Lee H.C., Raje N.S., Landgren O., Upreti V.V., Wang J., Avilion A.A., Hu X., Rasmussen E., Ngarmchamnanrith G., Fujii H. (2021). Phase 1 study of the anti-BCMA antibody-drug conjugate AMG 224 in patients with relapsed/refractory multiple myeloma. Leukemia.

[110] Tallman M.S., McDonald G.B., DeLeve L.D., Baer M.R., Cook M.N., Graepel G.J., Kollmer C. (2013). Incidence of sinusoidal obstruction syndrome following Mylotarg (gemtuzumab ozogamicin): A prospective observational study of 482 patients in routine clinical practice. Int. J. Hematol..

[111] Common Terminology Criteria for Adverse Events (CTCAE) v6.0. https://dctd.cancer.gov/research/ctep-trials/for-sites/adverse-events/ctcae-v6.pdf.

[112] Li J., Shen G., Liu Z., Liu Y., Wang M., Zhao F., Ren D., Xie Q., Li Z., Liu Z. (2023). Treatment-related adverse events of antibody-drug conjugates in clinical trials: A systematic review and meta-analysis. Cancer Innov..

[113] Khoury R., Saleh K., Khalife N., Saleh M., Chahine C., Ibrahim R., Lecesne A. (2023). Mechanisms of Resistance to Antibody-Drug Conjugates. Int. J. Mol. Sci..

[114] Firestone R.S., Socci N.D., Shekarkhand T., Zhu M., Qin W.G., Hultcrantz M., Mailankody S., Tan C.R., Korde N., Lesokhin A.M. (2024). Antigen escape as a shared mechanism of resistance to BCMA-directed therapies in multiple myeloma. Blood.

[115] Chen R., Herrera A.F., Hou J., Chen L., Wu J., Guo Y., Synold T.W., Ngo V.N., Puverel S., Mei M. (2020). Inhibition of MDR1 Overcomes Resistance to Brentuximab Vedotin in Hodgkin Lymphoma. Clin. Cancer Res..

[116] Linenberger M.L. (2005). CD33-directed therapy with gemtuzumab ozogamicin in acute myeloid leukemia: Progress in understanding cytotoxicity and potential mechanisms of drug resistance. Leukemia.

[117] Zhao Y., Short N.J., Kantarjian H.M., Chang T.C., Ghate P.S., Qu C., Macaron W., Jain N., Thakral B., Phillips A.H. (2024). Genomic determinants of response and resistance to inotuzumab ozogamicin in B-cell ALL. Blood.

[118] Mazor Y., Sachsenmeier K.F., Yang C., Hansen A., Filderman J., Mulgrew K., Wu H., Dall’Acqua W.F. (2017). Enhanced tumor-targeting selectivity by modulating bispecific antibody binding affinity and format valence. Sci. Rep..

[119] Zhao H., Atkinson J., Gulesserian S., Zeng Z., Nater J., Ou J., Yang P., Morrison K., Coleman J., Malik F. (2018). Modulation of Macropinocytosis-Mediated Internalization Decreases Ocular Toxicity of Antibody-Drug Conjugates. Cancer Res..

[120] Tsuchikama K., Anami Y., Ha S.Y.Y., Yamazaki C.M. (2024). Exploring the next generation of antibody-drug conjugates. Nat. Rev. Clin. Oncol..

[121] Gu Y., Wang Z., Wang Y. (2024). Bispecific antibody drug conjugates: Making 1+1>2. Acta Pharm. Sin. B.

[122] Hamblett K.J., Hammond P.W., Barnscher S.D., Fung V.K., Davies R.H., Hammond P.W., Hernandez A., Wickman G.R., Fung V.K., Ding T. (2019). ZW49, a HER2 targeted biparatopic antibody drug conjugate for the treatment of HER2 expressing cancers. Cancer Res..

[123] Knuehl C., Toleikis L., Dotterweich J., Ma J., Kumar S., Ross E., Wilm C., Schmitt M., Grote H.J., Amendt C. (2022). M1231 is a bispecific anti-MUC1xEGFR antibody-drug conjugate designed to treat solid tumors with MUC1 and EGFR co-expression. Cancer Res..

[124] Li J.Y., Perry S.R., Muniz-Medina V., Wang X., Wetzel L.K., Rebelatto M.C., Masson Hinrichs M.J., Bezabeh B.Z., Fleming R.L., Dimasi N. (2016). A Biparatopic HER2-Targeting Antibody-Drug Conjugate Induces Tumor Regression in Primary Models Refractory to or Ineligible for HER2-Targeted Therapy. Cancer Cell.

[125] DeVay R.M., Delaria K., Zhu G., Holz C., Foletti D., Sutton J., Bolton G., Dushin R., Bee C., Pons J. (2017). Improved Lysosomal Trafficking Can Modulate the Potency of Antibody Drug Conjugates. Bioconjug. Chem..

[126] Andreev J., Thambi N., Perez Bay A.E., Delfino F., Martin J., Kelly M.P., Kirshner J.R., Rafique A., Kunz A., Nittoli T. (2017). Bispecific Antibodies and Antibody-Drug Conjugates (ADCs) Bridging HER2 and Prolactin Receptor Improve Efficacy of HER2 ADCs. Mol. Cancer Ther..

[127] De Goeij B.E., Vink T., Ten Napel H., Breij E.C., Satijn D., Wubbolts R., Miao D., Parren P.W. (2016). Efficient Payload Delivery by a Bispecific Antibody-Drug Conjugate Targeting HER2 and CD63. Mol. Cancer Ther..

[128] Oberoi H.K., Garralda E. (2021). Unmasking New Promises: Expanding the Antigen Landscape For Antibody-Drug Conjugates. Clin. Cancer Res..

[129] Johnson M., El-Khoueiry A., Hafez N., Lakhani N., Mamdani H., Rodon J., Sanborn R.E., Garcia-Corbacho J., Boni V., Stroh M. (2021). Phase I, First-in-Human Study of the Probody Therapeutic CX-2029 in Adults with Advanced Solid Tumor Malignancies. Clin. Cancer Res..

[130] Singh S., Serwer L., DuPage A., Elkins K., Chauhan N., Ravn M., Buchanan F., Wang L., Krimm M., Wong K. (2022). Nonclinical Efficacy and Safety of CX-2029, an Anti-CD71 Probody-Drug Conjugate. Mol. Cancer Ther..

[131] American Association of Neurological Surgeons (AANS), American Society of Neuroradiology (ASNR), Cardiovascular and Interventional Radiology Society of Europe (CIRSE), Canadian Interventional Radiology Association (CIRA), Congress of Neurological Surgeons (CNS), European Society of Minimally Invasive Neurological Therapy (ESMINT), European Society of Neuroradiology (ESNR), European Stroke Organization (ESO), Society for Cardiovascular Angiography and Interventions (SCAI), Society of Interventional Radiology (SIR) (2018). Multisociety Consensus Quality Improvement Revised Consensus Statement for Endovascular Therapy of Acute Ischemic Stroke. Int. J. Stroke.

[132] Trang V.H., Zhang X., Yumul R.C., Zeng W., Stone I.J., Wo S.W., Dominguez M.M., Cochran J.H., Simmons J.K., Ryan M.C. (2019). A coiled-coil masking domain for selective activation of therapeutic antibodies. Nat. Biotechnol..

[133] Lyon R.P., Bovee T.D., Doronina S.O., Burke P.J., Hunter J.H., Neff-LaFord H.D., Jonas M., Anderson M.E., Setter J.R., Senter P.D. (2015). Reducing hydrophobicity of homogeneous antibody-drug conjugates improves pharmacokinetics and therapeutic index. Nat. Biotechnol..

[134] Kovtun Y.V., Audette C.A., Mayo M.F., Jones G.E., Doherty H., Maloney E.K., Erickson H.K., Sun X., Wilhelm S., Ab O. (2010). Antibody-maytansinoid conjugates designed to bypass multidrug resistance. Cancer Res..

[135] Zacharias N., Podust V.N., Kajihara K.K., Leipold D., Del Rosario G., Thayer D., Dong E., Paluch M., Fischer D., Zheng K. (2022). A homogeneous high-DAR antibody-drug conjugate platform combining THIOMAB antibodies and XTEN polypeptides. Chem. Sci..

[136] Su D., Zhang D. (2021). Linker Design Impacts Antibody-Drug Conjugate Pharmacokinetics and Efficacy via Modulating the Stability and Payload Release Efficiency. Front. Pharmacol..

[137] Chuprakov S., Ogunkoya A.O., Barfield R.M., Bauzon M., Hickle C., Kim Y.C., Yeo D., Zhang F., Rabuka D., Drake P.M. (2021). Tandem-Cleavage Linkers Improve the In Vivo Stability and Tolerability of Antibody-Drug Conjugates. Bioconjug. Chem..

[138] Satomaa T., Pynnonen H., Vilkman A., Kotiranta T., Pitkanen V., Heiskanen A., Herpers B., Price L.S., Helin J., Saarinen J. (2018). Hydrophilic Auristatin Glycoside Payload Enables Improved Antibody-Drug Conjugate Efficacy and Biocompatibility. Antibodies.

[139] Tao J., Gu Y., Zhou W., Wang Y. (2025). Dual-payload antibody–drug conjugates: Taking a dual shot. Eur. J. Med. Chem..

[140] Zhao Y., Ren P., Guan M., Tang J., Qi L., Fan X., Yin S., Lei G., Ke X. (2025). Abstract 1586: KH815, a novel dual-payload TROP2-directed antibody-drug conjugate, shows potent antitumor efficacy in pre-clinical tumor model. Cancer Res..

[141] Weidle U.H., Tiefenthaler G., Georges G. (2014). Proteases as activators for cytotoxic prodrugs in antitumor therapy. Cancer Genom. Proteom..

[142] Gregson S.J., Barrett A.M., Patel N.V., Kang G.D., Schiavone D., Sult E., Barry C.S., Vijayakrishnan B., Adams L.R., Masterson L.A. (2019). Synthesis and evaluation of pyrrolobenzodiazepine dimer antibody-drug conjugates with dual beta-glucuronide and dipeptide triggers. Eur. J. Med. Chem..

[143] Vaisitti T., Vitale N., Micillo M., Brandimarte L., Iannello A., Papotti M.G., Jaksic O., Lopez G., Di Napoli A., Cutrin J.C. (2022). Anti-CD37 alpha-amanitin-conjugated antibodies as potential therapeutic weapons for Richter syndrome. Blood.

[144] Pahl A., Lutz C., Hechler T. (2018). Amanitins and their development as a payload for antibody-drug conjugates. Drug Discov. Today Technol..

[145] Figueroa-Vazquez V., Ko J., Breunig C., Baumann A., Giesen N., Palfi A., Muller C., Lutz C., Hechler T., Kulke M. (2021). HDP-101, an Anti-BCMA Antibody-Drug Conjugate, Safely Delivers Amanitin to Induce Cell Death in Proliferating and Resting Multiple Myeloma Cells. Mol. Cancer Ther..

[146] Bekes M., Langley D.R., Crews C.M. (2022). PROTAC targeted protein degraders: The past is prologue. Nat. Rev. Drug Discov..

[147] Neumann C.S., Olivas K.C., Anderson M.E., Cochran J.H., Jin S., Li F., Loftus L.V., Meyer D.W., Neale J., Nix J.C. (2018). Targeted Delivery of Cytotoxic NAMPT Inhibitors Using Antibody-Drug Conjugates. Mol. Cancer Ther..

[148] Hicks S.W., Tarantelli C., Wilhem A., Gaudio E., Li M., Arribas A.J., Spriano F., Bordone R., Cascione L., Lai K.C. (2019). The novel CD19-targeting antibody-drug conjugate huB4-DGN462 shows improved anti-tumor activity compared to SAR3419 in CD19-positive lymphoma and leukemia models. Haematologica.

[149] Yu S.F., Lee D.W., Zheng B., Del Rosario G., Leipold D., Booler H., Zhong F., Carrasco-Triguero M., Hong K., Yan P. (2021). An Anti-CD22-seco-CBI-Dimer Antibody-Drug Conjugate (ADC) for the Treatment of Non-Hodgkin Lymphoma That Provides a Longer Duration of Response than Auristatin-Based ADCs in Preclinical Models. Mol. Cancer Ther..

[150] Drake P.M., Carlson A., McFarland J.M., Banas S., Barfield R.M., Zmolek W., Kim Y.C., Huang B.C.B., Kudirka R., Rabuka D. (2018). CAT-02-106, a Site-Specifically Conjugated Anti-CD22 Antibody Bearing an MDR1-Resistant Maytansine Payload Yields Excellent Efficacy and Safety in Preclinical Models. Mol. Cancer Ther..

[151] Abdelhamed S., Zhang X., Zeng W., Grogan B., Schilperoort L., James R., Burley K.C., Sarrett S.M., Bou L., Raha P. (2025). PF-08046032: A Novel, Investigational CD25-Directed Antibody-Drug Conjugate Optimized for Selective Depletion of Regulatory T Cells in Advanced Malignant Tumors. Mol. Cancer Ther..

[152] Ryan M., Lyski R., Bou L., Heiser R., Grogan B., Meyer D., Jin S., Simmons J., Conerly M., Senter P. (2020). SGN-CD30C, An Investigational CD30-Directed Camptothecin Antibody-Drug Conjugate (ADC), Shows Strong Anti Tumor Activity and Superior Tolerability in Preclinical Studies. Blood.

[153] Jeremy E., Artiga E., Elgamal S., Cheney C., Eicher D., Zalponik K., Orwick S., Mao C., Wasmuth R., Harrington B. (2025). CD37 in acute myeloid leukemia: A novel surface target for drug delivery. Blood Adv..

[154] Le Q., Tang T., Leonti A., Castro S., McKay C.N., Perkins L., Pardo L., Kirkey D., Hylkema T., Call L. (2023). Preclinical studies targeting CD74 with STRO-001 antibody-drug conjugate in acute leukemia. Blood Adv..

[155] Costanza M., Giordano C., von Brunneck A.C., Zhao J., Makky A., Vinh K., Montes-Mojarro I.A., Reisinger F., Forchhammer S., Witalisz-Siepracka A. (2025). Preclinical in vitro and in vivo evidence for targeting CD74 as an effective treatment strategy for cutaneous T-cell lymphomas. Br. J. Dermatol..

[156] Springer A.D., Wang R., Wang J., Du Q., Pi W., Nguyen A.Q., Li X., Khasanov A., Zhu T., Yan Z. (2024). Preclinical Evaluation of STI-8811, a Novel Antibody-Drug Conjugate Targeting BCMA for the Treatment of Multiple Myeloma. Cancer Res. Commun..

[157] Cardillo T.M., Govindan S.V., Zalath M.B., Rossi D.L., Wang Y., Chang C.H., Goldenberg D.M. (2018). IMMU-140, a Novel SN-38 Antibody-Drug Conjugate Targeting HLA-DR, Mediates Dual Cytotoxic Effects in Hematologic Cancers and Malignant Melanoma. Mol. Cancer Ther..

[158] Han Y.C., Jiang F., Piche-Nicholas N., Katragadda M., Prashad N., Charati M., Hu W., Leal M., Tumey N., Maderna A. (2018). Generation and preclinical characterization of CD123-CPI antibody-drug conjugate (ADC). Cancer Res..

[159] Chang C.A., Emberley E., D’Souza A.L., Zhao W., Cosgrove C., Parrish K., Mitra D., Payson E., Oleksijew A., Ellis P. (2024). ABBV-319: A CD19-targeting glucocorticoid receptor modulator antibody-drug conjugate therapy for B-cell malignancies. Blood.

[160] Hernandez-Ilizaliturri F.J., Kuruvilla J., Christian B.A., Flinn I.W., Assouline S.E., Ulrickson M.L., Landsburg D.J., Stuart M., Lowman H., Levin N. (2022). Results from a phase I pharmacokinetic (PK) and safety study of TRPH-222, a novel CD22-targeting antibody-drug conjugate, in patients with relapsed/refractory B-cell non-Hodgkin lymphoma (R/R NHL). Hemasphere.

[161] Zammarchi F., Havenith K.E., Sachini N., Janghra N., Chivers S., Idusogie E., Gaudio E., Tarantelli C., Bertelli F., Santos K. (2024). ADCT-602, a Novel PBD Dimer-containing Antibody-Drug Conjugate for Treating CD22-positive Hematologic Malignancies. Mol. Cancer Ther..

[162] Spriano F., Tarantelli C., Cascione L., Gaudio E., Golino G., Scalise L., Cacciapuoti M.T., Zucca E., Stathis A., Van Berkel P.H. (2024). Targeting CD25+ lymphoma cells with the antibody-drug conjugate camidanlumab tesirine as a single agent or in combination with targeted agents. Br. J. Haematol..

[163] Hamadani M., Collins G.P., Caimi P.F., Samaniego F., Spira A., Davies A., Radford J., Menne T., Karnad A., Zain J.M. (2021). Camidanlumab tesirine in patients with relapsed or refractory lymphoma: A phase 1, open-label, multicentre, dose-escalation, dose-expansion study. Lancet Haematol..

[164] Herrera A.F., Ansell S.M., Zinzani P.L., Radford J., Maddocks K., Pinto A., Collins G.P., Bachanova V., Bartlett N.L., Bence-Bruckler I. (2025). Camidanlumab tesirine for relapsed or refractory classic Hodgkin lymphoma: A phase 2 study. Blood Adv..

[165] Shen Y., Yang T., Cao X., Zhang Y., Zhao L., Li H., Zhao T., Xu J., Zhang H., Guo Q. (2019). Conjugation of DM1 to anti-CD30 antibody has potential antitumor activity in CD30-positive hematological malignancies with lower systemic toxicity. mAbs.

[166] Deckert J., Park P.U., Chicklas S., Yi Y., Li M., Lai K.C., Mayo M.F., Carrigan C.N., Erickson H.K., Pinkas J. (2013). A novel anti-CD37 antibody-drug conjugate with multiple anti-tumor mechanisms for the treatment of B-cell malignancies. Blood.

[167] Stathis A., Flinn I.W., Madan S., Maddocks K., Freedman A., Weitman S., Zucca E., Munteanu M.C., Lia Palomba M. (2018). Safety, tolerability, and preliminary activity of IMGN529, a CD37-targeted antibody-drug conjugate, in patients with relapsed or refractory B-cell non-Hodgkin lymphoma: A dose-escalation, phase I study. Investig. New Drugs.

[168] Levy M.Y., Jagadeesh D., Grudeva-Popova Z., Trněný M., Jurczak W., Pylypenko H., André M., Nasta S.D., Rechavi-Robinson D., Toffanin S. (2021). Safety and Efficacy of CD37-Targeting Naratuximab Emtansine PLUS Rituximab in Diffuse Large B-Cell Lymphoma and Other NON-Hodgkin’S B-Cell Lymphomas—A Phase 2 Study. Blood.

[169] Cole F.M., Laszlo G.S., Lunn-Halbert M.C., Kehret A.R., Zweidler-McKay P.A., Rodriguez-Arboli E., Wu D., Nyberg K., Li J., Lim S.Y.T. (2025). Preclinical characterization of the anti-leukemia activity of the CD123 antibody-drug conjugate, pivekimab sunirine (IMGN632). Leukemia.

[170] Daver N.G., Montesinos P., DeAngelo D.J., Wang E.S., Papadantonakis N., Todisco E., Sweet K.L., Pemmaraju N., Lane A.A., Torres-Minana L. (2024). Pivekimab sunirine (IMGN632), a novel CD123-targeting antibody-drug conjugate, in relapsed or refractory acute myeloid leukaemia: A phase 1/2 study. Lancet Oncol..

[171] Dutta D., Pan P., Fleming R., Andrade-Campos M., Belova E., Wheeler J., Cheung P., Santacroce P., Daniels C., Sabol D. (2023). First Disclosure of AZD9829, a TOP1i-ADC Targeting CD123: Promising Preclinical Activity in AML Models with Minimal Effect on Healthy Progenitors. Blood.

[172] Maragno A.-L., Seiss K., Newcombe R., Mistry P., Schnell C.R., Von Arx F., Koenig J., Malamas A.S., Le Toumelin-Braizat G., Bresson L. (2024). S227928: A Novel Anti-CD74 ADC with MCL-1 Inhibitor Payload for the Treatment of Acute Myeloid Leukemia (AML) and Other Hematologic Malignancies. Blood.

[173] Abrahams C.L., Li X., Embry M., Yu A., Krimm S., Krueger S., Greenland N.Y., Wen K.W., Jones C., DeAlmeida V. (2018). Targeting CD74 in multiple myeloma with the novel, site-specific antibody-drug conjugate STRO-001. Oncotarget.

[174] Li X., Abrahams C., Yu A., Embry M., Henningsen R., DeAlmeida V., Matheny S., Kline T., Yam A., Stafford R. (2023). Targeting CD74 in B-cell non-Hodgkin lymphoma with the antibody-drug conjugate STRO-001. Oncotarget.

[175] Vaisitti T., Arruga F., Vitale N., Lee T.T., Ko M., Chadburn A., Braggio E., Di Napoli A., Iannello A., Allan J.N. (2021). ROR1 targeting with the antibody-drug conjugate VLS-101 is effective in Richter syndrome patient-derived xenograft mouse models. Blood.

[176] Li F., Li Y., Zhang L., Zhang C.K., Hu H.-H.A., Zhang J., Pan Y., Jung J., Lee S.H., Ryu H.-M. (2021). CS5001, a novel ROR1-targeting antibody drug conjugate (ADC) armed with tumor-cleavable β-glucuronide linkers and pyrrolobenzodiazepine (PBD) prodrugs for hematological and solid malignancies. Mol. Cancer Ther..

[177] Lemech C.R., Zuniga R., Barve M.A., Song Y., Zhang J., Zhou K., Zhang L., Shen L., Bishnoi S., Cherng H.-J.J. (2024). A phase 1a/b, multi-regional, first-in-human study of CS5001, a novel anti-ROR1 ADC, in patients with advanced solid tumors and lymphomas. J. Clin. Oncol..

[178] Chakraborty R., Yan Y., Royal M. (2021). A Phase 1, Open-Label, Dose-Escalation Study of the Safety and Efficacy of Anti-CD38 Antibody Drug Conjugate (STI-6129) in Patients with Relapsed or Refractory Multiple Myeloma. Blood.

[179] Saggu G., Lee M.Y., Dunbar F., Singh R., Sachsenmeier K., Vogl D.T., Atrash S., Holstein S.A., Nadeem O., Kaufman J.L. (2023). Modakafusp Alfa Demonstrates Type I Interferon-Mediated Innate and Adaptive Immune Enhancement in a Phase 1/2 Study in Patients with Relapsed and/or Refractory Multiple Myeloma (RRMM). Blood.

[180] Vogl D.T., Atrash S., Holstein S.A., Nadeem O., Benson D., Chaudry M., Biran N., Suryanarayan K., Li C., Liu Y. (2025). Targeted interferon therapy with modakafusp alfa for relapsed or refractory multiple myeloma. Blood.

[181] Holstein S.A., Atrash S., Mian H., Dimopoulos M.A., Schjesvold F., Popat R., Shah N., Gatt M.E., Gocke C.B., Frenzel L. (2025). A phase 2 randomized study of modakafusp alfa as a single agent for patients with relapsed/refractory multiple myeloma. Blood.

[182] VanWyngarden M.J., Walker Z.J., Su Y., Perez de Acha O., Stevens B.M., Forsberg P.A., Mark T.M., Matsui W., Liu B., Sherbenou D.W. (2023). CD46-ADC Reduces the Engraftment of Multiple Myeloma Patient-Derived Xenografts. Cancers.

[183] Ailawadhi S., Kelly K.R., Vescio R.A., Jagannath S., Wolf J., Gharibo M., Sher T., Bojanini L., Kirby M., Chanan-Khan A. (2019). A Phase I Study to Assess the Safety and Pharmacokinetics of Single-agent Lorvotuzumab Mertansine (IMGN901) in Patients with Relapsed and/or Refractory CD-56-positive Multiple Myeloma. Clin. Lymphoma Myeloma Leuk..

[184] Jagannath S., Heffner L.T., Ailawadhi S., Munshi N.C., Zimmerman T.M., Rosenblatt J., Lonial S., Chanan-Khan A., Ruehle M., Rharbaoui F. (2019). Indatuximab Ravtansine (BT062) Monotherapy in Patients With Relapsed and/or Refractory Multiple Myeloma. Clin. Lymphoma Myeloma Leuk..

[185] Kelly K.R., Ailawadhi S., Siegel D.S., Heffner L.T., Somlo G., Jagannath S., Zimmerman T.M., Munshi N.C., Madan S., Chanan-Khan A. (2021). Indatuximab ravtansine plus dexamethasone with lenalidomide or pomalidomide in relapsed or refractory multiple myeloma: A multicentre, phase 1/2a study. Lancet Haematol..

